# A Review on the Corrosion Behaviour of Nanocoatings on Metallic Substrates

**DOI:** 10.3390/ma12020210

**Published:** 2019-01-10

**Authors:** Dana H. Abdeen, Mohamad El Hachach, Muammer Koc, Muataz A. Atieh

**Affiliations:** 1 Sustainable Development Division, College of Science and Engineering, Hamad Bin Khalifa University, P.O. Box 34110, Doha, Qatar; DanAbdeen@hbku.edu.qa (D.H.A.); mohammadelhachach@gmail.com (M.E.H.); mkoc@qf.org.qa (M.K.); 2 Department of Chemical Engineering, College of Engineering, Qatar University, P.O. Box 2713, Doha, Qatar; 3 Qatar Environment and Energy Research Institute (QEERI), Hamad Bin Khalifa University, P.O. Box 5825, Doha, Qatar

**Keywords:** corrosion, nanocoating, metallic nanocoating, ceramic nanocoating, nanocomposite coating, corrosion factors

## Abstract

Growth in nanocoatings technology is moving towards implementing nanocoatings in many sectors of the industry due to their excellent abilities. Nanocoatings offer numerous advantages, including surface hardness, adhesive strength, long-term and/or high-temperature corrosion resistance, the enhancement of tribological properties, etc. In addition, nanocoatings can be applied in thinner and smoother thickness, which allows flexibility in equipment design, improved efficiency, lower fuel economy, lower carbon footprints, and lower maintenance and operating costs. Nanocoatings are utilised efficiently to reduce the effect of a corrosive environment. A nanocoating is a coating that either has constituents in the nanoscale, or is composed of layers that are less than 100 nm. The fine sizes of nanomaterials and the high density of their ground boundaries enable good adhesion and an excellent physical coverage of the coated surface. Yet, such fine properties might form active sites for corrosion attack. This paper reviews the corrosion behaviour of metallic, ceramic, and nanocomposite coatings on the surface of metallic substrates. It summarises the factors affecting the corrosion of these substrates, as well as the conditions where such coatings provided required protection.

## 1. Introduction 

**Figure 1:**
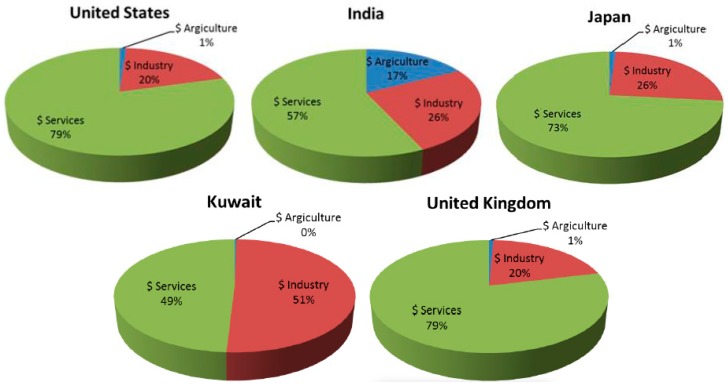
Corrosion cost of five different countries per economic sector as indicated by International Measures of Prevention, Application, and Economics of Corrosion Technology (IMPACT) study, a NACE international report [4].

Corrosion is one of the major research areas that has been attracting the attention of researchers for over 150 years, since it is recognised as a problem causing degradation, failure, and serious accidents and hazards in many industrial processes and domestic systems [1,2]. Corrosion is the deterioration of the metals due to their reaction with a corrosive element in their surroundings, such as chlorine, fluorine, carbon dioxide, oxygen, etc. Damages due to corrosion in terms of economic aspects include repair and maintenance costs, loss of materials, damage to equipment, a decrease in efficiency, and loss of useful or productive life. Furthermore, corrosion damages have other social effects, such as safety impacts (cause of fire, explosions, release of toxic products), health impacts (personal injuries, pollution due to contamination of toxic products), the depletion of resources, etc. [3]. A National Association in Corrosion Engineers (NACE) study estimated the global cost of corrosion to be $255 billion USD, which accounts for 3.4% of the global gross domestic product (GDP) [4]. In the United States (U.S.) economy, the direct and indirect annual costs of corrosion estimated to be $552 billion, which weighs for 6% of the its GDP [5]. The direct effects of corrosion include the cost of controlling and repairing the damages incurred by household appliances, highway bridges, automobiles, airplanes, industrial plants such as energy production and distribution systems, petrochemical, desalination, pharmaceutical, etc. Other indirect corrosion costs are as substantial as the direct ones, and can be related to the loss in productivity due to delays, failures, or outages, as well as taxes and the overhead of corrosion cost, etc. The cost of corrosion for the economic sector for five different regions were collected, as shown in Figure 1. An analysis showed that the United States, United Kingdom, and Japan had similar corrosion costs related to advanced industries and services economies, whereas India and Kuwait had substantial contribution from the agricultural and oil industry economies, respectively [4]. Hence, proper corrosion prevention, monitoring, and applying safety standards and practices in these categories can save 15–35% of the losses caused by corrosion [4].

**Figure 2:**
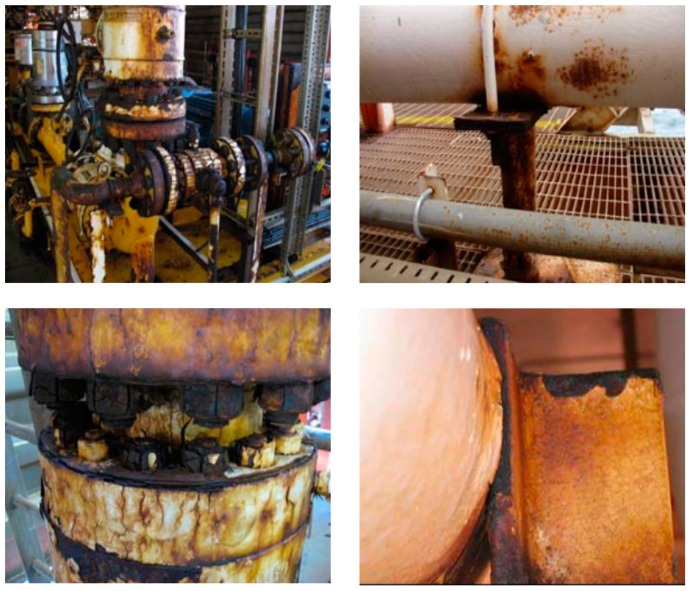
Example of corrosion effect on bolts, valves, flanges, piping, and pipe support [7].

Corrosion is a natural process that causes the dissolution of a material in the presence of aggressive environments. The most important factors that affect the occurrence of corrosion depend on the material and the environmental conditions. The material corrodes if it is active or adjacent to a nobler material in the galvanic series, which causes the dissolution of the first one. Specific environmental conditions make the material susceptible to corrosion, such as dissolved gases (mainly oxygen and carbon dioxide), temperature, pH, tensile stresses, and cyclic 2 stresses. Corrosion can happen in different forms, depending on the mechanism of corrosion. These can include: uniform, galvanic, crevice, pitting, environmentally-induced cracking, intergranular, dealloying, and erosion corrosion. Uniform corrosion is the form with the most incidences, and the highest tonnage of metal waste. While the others are localised corrosion, and might not consume a lot of material, they are difficult to predict and control, and might undertake an early unnoticeable failure [6]. Unless good practices are followed in the field, corrosion in all of its forms can cause dramatic failures in major parts of any processes such as bolts, flanges, pipes, etc., as shown in Figure 2 [7]. 

Corrosion prevention is performed through different techniques, and choosing the right one should be done while optimising between process cost, process performance, and corrosion effects. Corrosion can be prevented by: (a) *Material selection*, where the material is either relatively unreactive in the galvanic series or can form a protective oxide layer (passivate) in a particular environment; (b) *Adjusting the environment conditions*, such as the addition of inhibitors [8,9], adjusting the pH and temperature of the surroundings, reducing the sulphur [10], oxygen, and chloride content [11], lowering the flow velocity, cleaning from sand and sediments, etc.; (c) *Surface modifications*, which is achieved by applying physical barriers such as films and coatings to reduce crevices and cracks [12,13]; (d) *Cathodic protection*, where the corrosion current is suppressed and is forced to flow to the metal to be protected. It is achieved by using a power source or attaching a more active (anodic) material to the structure to be protected [14]. Each protection method has its own advantages and disadvantages, and choosing the right one depends on the requirements of the operational conditions of the system under consideration. 

Coating is the most widely used method for preventing, minimising, or controlling corrosion due to an available and possible variety of coating materials and coating processes for different conditions and applications. Coating, either of inner or outer surfaces, can be applied within different temperature ranges; it even provides an additional gain of smoother surfaces that enhances the efficiency of the interface and flow on surfaces [15]. Applying a coating might have a high cost, but it is considered to be more feasible in the long term and on the large-scale applications, as it provides tremendous savings in terms of maintenance cost, natural resources, safety, risk of equipment faults, the life of the equipment, etc. [16]. In general, coatings reduce corrosion by providing passive [17] or active protection [18]. Passive protection is obtained when the coating forms a physical barrier of oxides between the substrate and the surrounding environment [19]. Active protection is obtained when chemicals (inhibitors) are added to aggressive environments to prevent or minimise corrosion. Inhibitors minimise the corrosion rate by either being chemically absorbed on the surface of the metal and forming a protective thin film over it, or by reacting with the corrosive component in the aqueous media [20].

Recently, nanomaterials have been introduced as an effective technique to reduce corrosion. Nanomaterials are materials that have at least one of their morphological features such as grain size, particle size, structure size, etc., in the nanoscale (less than 100 nm) [18]. They can be of zero dimension (nanoparticles), one dimension (nanotubes, nanowires, and nanorods), or two dimensions (nanoplatelet, nanosheets, and nanofilms). Nanomaterials possess improved thermal, mechanical, physical, chemical, magnetic, electronic, and optical properties [21]. This is primarily due to their small sizes, which allow higher volume fractions at the surfaces and thus higher interaction areas [22]. Nanomaterials are considered to have promise in the reduction of the corrosion rate of the metal substrates through surface modification with coatings that have nanocrystalline structures. 

## 2. Nanocoating and Its Role in Corrosion Prevention

A nanocoating is an ultrafine microstructure where all of the constituents (boundaries, crystals, phases, etc.) are on the scale of less than 100 nm. These coatings can also be built up by layers that are thinner than 100 nm [23,24]. They have a high density of grain boundaries, interphase boundaries, dislocations, etc., where the spacing between them approaches interatomic distances. Therefore, nanostructured coatings exhibit different properties from the larger-grained, conventional coatings, which enabled them to overcome the mechanical and corrosion properties of their counterparts. Nanocoatings can be classified according to the constituent materials, such as metallic and ceramic nanocoatings. They can also be composed of two or more materials that are in the nanoscale, as in nanocomposite coatings. 

Nanocoating has one component that is in the nanoscale. Due to the very fine sizes of the particles used in this nanocoating, filling the spaces and blocking the corrosive elements from diffusing into the surface of the substrate will be more efficient. In addition, the high density of the nanocoatings’ grain boundaries provides better adhesion properties, which will increase the lifetime of the coating [6]. Nanocoatings provide superior mechanical and electronic properties, which make them stronger, harder [25], and have better resistance to environments with corrosion and wear [26]. Nanocoating technology has influenced the development of paints greatly with the addition of properties such as self-healing [27], self-cleaning [26], and high scratch and wear resistance [28]. It also enabled the availability of replacements for chromium toxic coating [29,30]. In the same manner, smart nanocoatings greatly benefit in reducing corrosion and biofouling effects. They are developed to respond to external stimulus such as pH, humidity, heat, stress, coating distortion, electromagnetic radiation, etc., by releasing controlled amounts of inhibitors in order to repair and cure defects and damages [9,31]. Traditional coatings of microsized particles or thicknesses would have a different corrosion behaviour than that of nanocoatings [32]. For example, a using zinc coating of nanothickness overcomes the problems of poor weldability and difficulty in achieving specular finish after painting [32].

Due to the extraordinary properties that the nanocoating possess, they are used in everyday practise such as clothing, computers, cell phones, eyeglasses, etc. In the building field, they are used in tiles, windows, flooring, walls, paints, air filters, etc. The utilisation of the nanolayer in these appliances makes them flame-retardant, wear and scratch resistant, anti-graffiti, corrosion resistant, self-cleaning, and electrically conductive. They also have good adherence, optical clarity, anti-fogging and anti-fouling properties, and are suitable as a photovoltaic material [33,34,35]. In the biomedical field, metallic nanocoatings are used to modify surface properties when needed. They are used in the medicine industry primarily for etch protection, surface coverage, and anti-corrosion functions, in addition to other secondary functions such as drug delivery and biocompatibility [36]. For all of the mentioned properties that the nanocoatings hold, they are used in many other fields such as the military, the automobile industry, energy efficiency, the environment, etc. 

**Figure 3:**
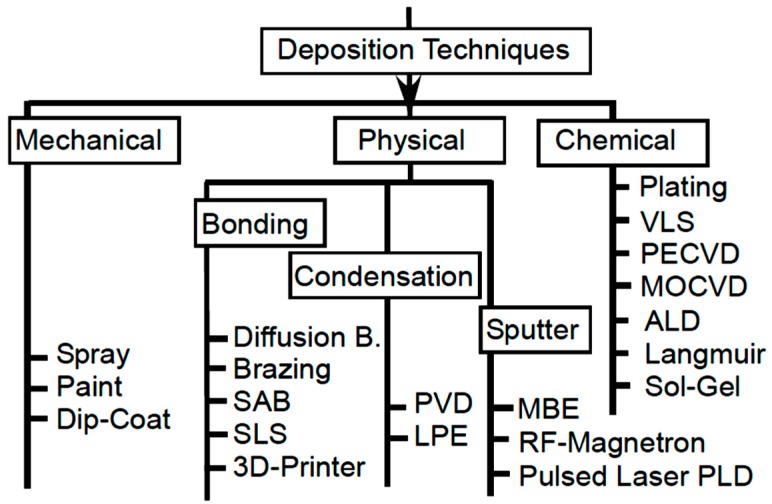
Thin film deposition methods [37].

Nanocoatings can be obtained by three general deposition methods, as shown in Figure 3: mechanical, physical, and chemical deposition [37]. Mechanical deposition is the cheapest, and can be achieved through spray, paint, spin-coating, or dip-coating. Physical deposition can be done by either bonding, condensation, or sputtering. In physical diffusion bonding, a moderate pressure and temperature are applied, while in brazing bonding, lubricants are added under higher temperatures. Bonding with surface-activated bonding (SAB) is performed at low temperatures and pressures for cleaned and atomically flat-polished surfaces. Moreover, selective laser sintering (SLS) is a three-dimensional (3D) printing technique in which a material is built layer-by-layer with the new manufacturing technology, additive manufacturing. The second method of physical bonding is condensation. It is usually performed at vacuum as in physical vapour deposition (PVD), whereas it might be performed at regular pressure conditions, such as in liquid phase epitaxy (LPE). Sputtering techniques are usually more expensive as they have lower growth rates, yet they produce perfect epitaxial growth with strong bonds. Sputtering is conducted with either molecular beam epitaxy (MBE), radio frequency (RF) magnetron, or pulsed laser deposition (PLD). Finally, chemical bonding techniques are usually cheaper, but requires expensive precursors, such as in Langmuir, sol-gel, and atomic layer deposition (ALD). Plasma-enhanced magneto optical chemical vapour deposition works at specified substrate temperature, pressure, and power [37]. The graph in Figure 3 is mostly incomplete, and there is more than can be added to it. 

Each of the above-mentioned techniques of thin film deposition on the surface of the substrate affect the uniformity and surface properties such as strength, fracture toughness, and ductility [38]. Each technique has its own cons and pros, and choosing the matching technique should be done while studying all of the processing elements. As in the case with conventional coatings, the technique should be applied with the optimised conditions in order to achieve the best surface coverage of the nanocoating in terms of uniformity, smoothness, adhesion, crack-free surfaces, etc. For example, dip coating is inexpensive and can coat complex shapes, but it might suffer from thermal expansion mismatch and require high sintering temperatures. Pulsed laser deposition and hot pressing can produce dense and uniform coatings, but they have the same disadvantages as dip-coating. Some techniques might produce an amorphous structure due to the rapid cooling, as in thermal spraying and sputter-coating. Sol-gel is a preferred technique, as it has low pressing temperatures and it is a relatively cheap coating; however, it requires expensive raw materials, and needs a controlled processing atmosphere [39]. Hence, more studies should be conducted to optimise the conditions and processing steps of different nanocoating techniques, as multi-step technologies are not attractive in the industrial market. 

Nanocoatings might not function as protective surfaces in some circumstances. A nanocoating is an effective physical barrier at high-temperature applications, as the high density of their grain boundaries provide fast diffusion paths of passivated ions and better adhesion of the protective oxide layer to the substrate’s surface [40]. Yet, the higher grain boundary fraction provides more anodic sites, which makes the surface more susceptible to corrosion attack. Moreover, nanocoatings form a defensive structure by incorporating in the vacancies, dislocations, and grain/interphase boundaries. These features have the advantage of forming a more effective passivation layer, as the diffusion of passivating ions will be faster. On the other hand, the agglomeration of these nanosized materials might happen due to the accelerated diffusion of aggressive ions, which causes non-uniform surfaces and increases the possibility of active sites formation, thus decreasing corrosion resistance [18]. Such a contradiction urges the need to study the corrosion behaviour of each nanocoating, while taking into consideration all of the surrounding conditions that are involved. 

**Figure 4:**
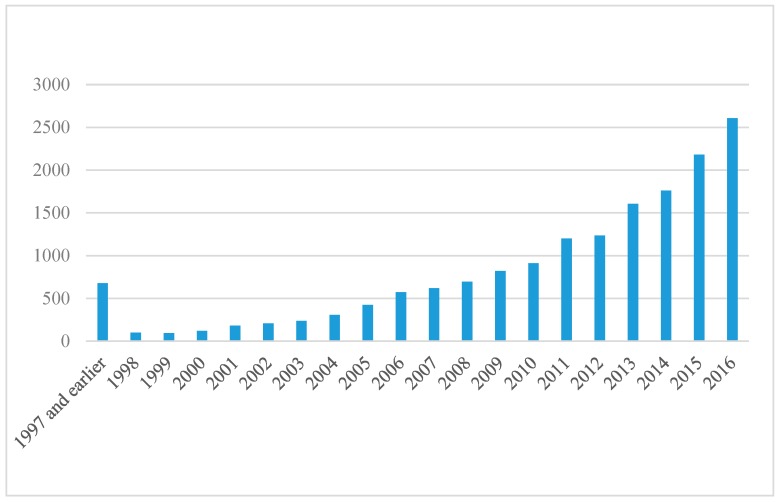
Number of published papers in the field of nanocoating and corrosion.

**Figure 5:**
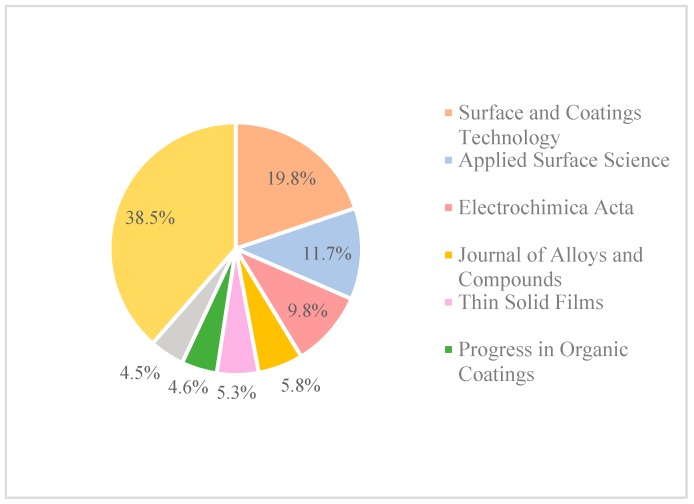
Distribution of published papers in nanocoating and corrosion among different journals.

Up until now, the wear/scratch resistance and corrosion behaviour of nanocoated surfaces is still under investigation; more research needs to be performed in this area. According to statistics from a ScienceDirect Journal search, research on the corrosion of nanocoatings started to gain more interest in 1998. Since then, the published papers related to nanocoating and corrosion have been increasing, but in a limited trend, as the maximum number of papers that have been published that are related to this field is around 2500 papers, as Figure 4 shows. The Surface and Coatings Technology, Applied Surface Science, and Electrochimica Acta journals have the highest percentages of papers that are published in this area (Figure 5). 

In the current paper, the corrosion behaviour of different nanocoatings is introduced by presenting some corrosion testing conducted on these nanocoatings under specific conditions. Each test can measure some corrosion parameters and help with understanding the corrosion mechanism for the tested sample. Immersion tests measure the weight loss of an immersed coupon after being exposed to aqueous solution for a certain time. It is intended to be used for long-term examinations and whenever uniform corrosion mechanism is expected to occur [6]. On the other hand, a potentiodynamic test is an electrochemical test where potential is applied at a specific scan rate and the current density is measured. The obtained current is due to the oxidation–reduction reactions happening on the surface of the metal, which provides the corrosion rate of the sample and the passivation behaviour from the potential versus the current curve. A potentiodynamic scan is used to identify corrosion parameters whenever localised corrosion forms, such as pitting and crevice corrosion [1]. An electron impedance spectroscopy (EIS) test is another electrochemical test that measures the electrochemical impedance, which indicates the ability of the cell to resist current flow. The impedance is measured by applying a sinusoidal potential to the cell, measuring the current as a response of this applied potential. Impedance spectra can be represented by Bode and Nyquist plots, which can provide information about different resistances in the cell and the controlling mechanisms in corrosion reactions. An equivalent electrical circuit model representing the tested cell can also be obtained with identifying resistances in the cell, such as charge transfer resistance, electrolyte resistance, coating resistance, polarisation resistance, etc. [41].

The corrosion rate can be represented by the penetration rate, which is the thickness loss of the material per unit of time. The corrosion rate can be expressed by different units such as mpy (mils per year, 1 mil = 0.0254 mm), mm/yr, µm/yr, etc. For typical ferrous and nickel alloys, the relative corrosion resistance of a metal can be categorised according to the corrosion rate value as follows (corrosion rate values in brackets are in mm/yr); outstanding corrosion resistance (<0.02), excellent (0.02–0.1), good (0.1–0.5), fair (0.5–1), poor (1–5), and unacceptable (5+). Rates greater than 5 mpy to 200 mpy are usually excessive for more expensive alloys, while rates above 200 mpy are sometimes acceptable for cheaper materials (e.g., cast iron) of a thicker cross-section [6]. 

The corrosion behaviour of nanocoatings is affected by different factors such as the environment, the substrate, the nanocoating composition, etc. The following sections discuss these factors in detail for different kinds of nanocoatings that were categorised according to the nanocoating material: metallic, ceramic, and nanocomposite coatings. At the end, some conclusions and recommendation for future work to be done in this area are presented. 

### 2.1. Metallic Nanocoating

Metallic nanocoating includes one or more of the pure metals such as Cadmium (Cd), Nickel (Ni), Tungsten (W), Zinc (Zn), Phosphorous (P), Cobalt (Co), Iron (Fe), Cupper (Cu), etc. Nanocoating can be of a pure metal [32,42,43], or alloyed for purposes of the enhancement of properties. Such enhancement is reinforced with the utilisation of nanosized coating, as nanomaterials behave differently than micromaterials [44]. Metallic nanocoatings can be produced through more than one technique such as sputtering [45] and multi-arc ion plating [46], in addition to electrodeposition, which was shown to be the most used technique in depositing metals [32,42,44,47,48,49,50,51]. They have a wide range of applications in many areas, such as automotive, aerospace [26], seawater condensers and tubes [44], electronic industries, water electrolysis [48], energy generation [52], etc. 

The corrosion behaviour of metallic nanocoatings involves different factors, which contribute in an individual or combined effect. The most influential factors are introduced in the below section. 

#### 2.1.1. Nanocoating Composition

Introducing metals in the nanocoating improves their physical [53], chemical [32], mechanical [26], and thermal [30] properties. Producing the metallic coating in the nanoscale accomplishes either protection for the coated substrate or tremendous enhancement of any of the coating’s properties. For example, a thinner nanocoating of zinc overcomes both the weldability and surface finish problems that arise when the thickness of the zinc coating needs to be increased to provide the required protection [32]. However, the corrosion resistance of pure zinc is insufficient at high temperatures and severe oxidising conditions; hence, it is alloyed with metals to provide higher corrosion resistance. Zn–Ni alloy coatings have been shown to have higher hardness than both zinc and cadmium separately [32]. In addition, Zn–Ni alloys are leading candidates for replacing cadmium in aerospace applications, as the latter does not have enough adhesive wear resistance [26]. Another example for efficient nanocoating is nanocrystalline cobalt and its alloys. They have been identified as an economic wear and corrosion resistance replacement for hard and toxic chromium [54,55]. 

Alloyed metal provides superior properties compared to pure metal, even at the nanoscale. An nanocrystalline Ni–W alloy has better hardness and scratch resistance compared to pure nanocrystalline W [25]. Alloying nickel with copper increases the corrosion resistance of nickel under a reducing environment and decreases pitting in seawater [44]. Moreover, alloying nickel with phosphorous makes the coating easier to passivate under acidic environments than pure Ni [56]. 

**Figure 6:**
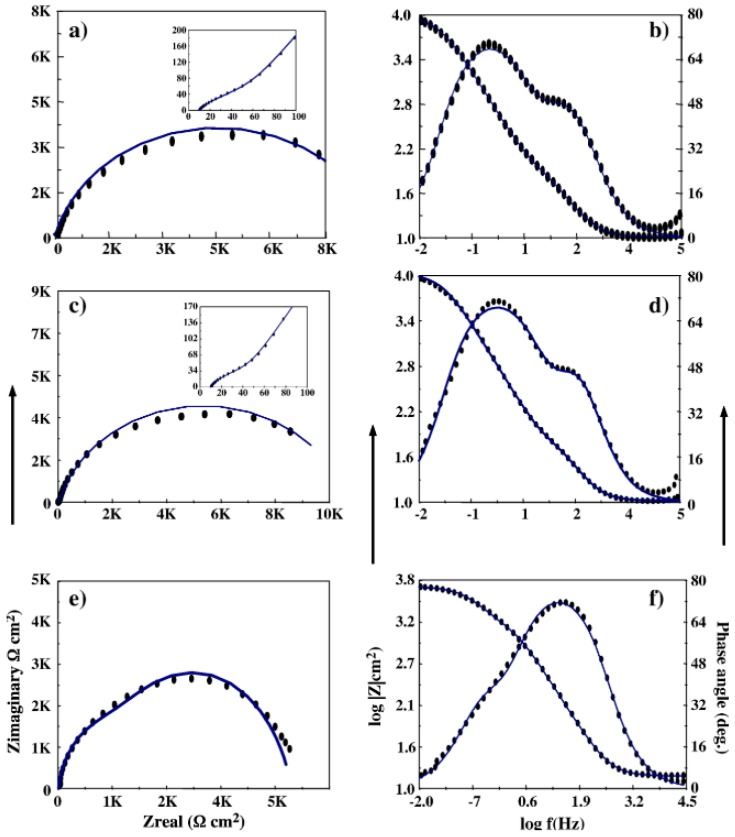
Nyquist and Bode plots for pulse deposited cobalt–phosphorous (Co–P) coating on mild steel substrate: (**a**,**b**) Co–P (7 wt.% P); (**c**,**d**) Co–P (9 wt.% P); and (**e**,**f**) Co–P (12 wt.% P) [49].

An electrodeposited nanocrystalline Zn–Ni nanocoating was tested in NaCl solution, and found that 13.31 wt.% (26-nm grain size) [52] and 17.62 wt.% (37-nm grain) [48] of Ni content achieved the best corrosion resistance. The addition of phosphorous was shown to improve the corrosion resistance of nanocrystalline Co–Ni alloy coatings in 3.5 wt.% NaCl solution for both pulse and direct current electrodeposited samples. A direct deposition of an intermediate concentration of 9% of the phosphorus (P) achieved the best corrosion behaviour, as shown in Figure 6. The impedance spectra obtained from Nyquist plots (Figure 6a–c) showed that the film resistance (R_f_) is higher for a higher P content (R_f_ is part of overall resistance of the coating). However, measured double-layer capacitance (Q_dl_) and coating capacitance (Q_coat_) obtained from Bode plots (Figure 6b,c,e), found that P content lower than 9 wt.% indicated better surface homogeneity. Hence, although higher P content improved the film resistance, it had protrusions that tended to decrease the overall coating resistance [49]. Longfei et al. [57] showed that varying the percentage of phosphorous in Ni–P nanocoating from 5 wt.% to 15 wt.% transitions the alloy structure from crystalline to an amorphous structure. Increasing the phosphorous content in the Ni–P nanocoating increases the corrosion resistance in acidic and neutral media, while this resistance will not improve in alkaline media when increasing phosphorous concentrations. The homogenous amorphous structure of phosphorous proved to have better resistance to chloride ions attack than the crystallised structure, since only the amorphous structure passivates in acidic and neutral solutions [57]. Moreover, an addition of 1.1% of phosphorous had a superior effect over the fine grain size in acidic media. Nanocrystalline Co-1.1 wt.% P alloy coating (average grain size: 10 nm) shifted the corrosion potential value by 59 mV to the positive side with respect to the nanocrystalline Co (average grain size: 20 nm) and microcrystalline Co when electron impedance spectroscopy (EIS) testing was conducted in a deaerated 0.1-M H_2_SO_4_ solution [58]. 

#### 2.1.2. Coating Structure Size

**Figure 7:**
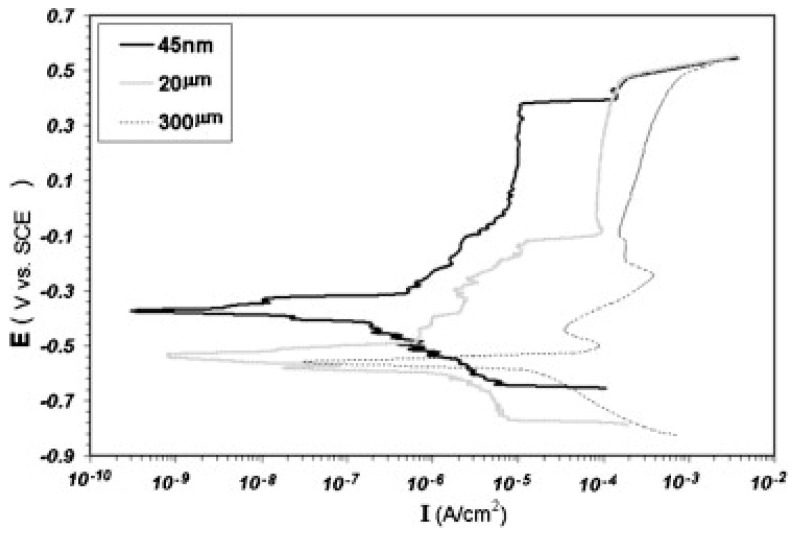
Effect of nanocoating and microcoating structure size. Nanograin size had the lowest i_corr_ in 10 wt.% NaOH [53].

The corrosion rate of the pulse–current electrodeposition of nanocrystalline zinc was 60% lower than the microcrystalline electrogalvanised steel samples (EG) when tested in NaOH solution. Examinations showed a complete coverage of the nanocrystalline zinc protective film, which was attributed to the nanocrystalline structure, and enhanced both the kinetics of passivation and the stability of the passive film [32]. Other research work on an nanocrystalline Ni–Cu alloy coating agreed that the nanocrystalline structure gave better corrosion resistance than the microcrystalline one, since a higher grain boundary density in the nanocrystalline structure speeded up the formation of a stable and protective oxide layer [44,48]. Figure 7 shows that the same results were obtained when nanocrystalline iron was pulse deposited on low-carbon steel samples and tested in alkaline solutions. It had better corrosion resistance than microcrystalline samples such as cast and annealed iron, which had grain sizes of 20 µm and 500 µm, respectively [53]. 

This was not in agreement with the work of Aledresse and Alfantazi, which showed a better corrosion resistance of nanocrystalline cobalt (67 nm) over polycrystalline cobalt (100 µm) in alkaline media, but a higher corrosion current density. This indicates that finer grain sizes of the coating might provide defect sites at the grain boundaries and triple junctions, which makes the corrosion easier to initiate at these active sites. Nanocrystalline structures have a higher volume fraction of the intercrystalline constituents—more than the polycrystalline structure—and thus more active sites are available in the nanocrystalline structure [54]. 

#### 2.1.3. Nanocoatings’ Grain Size

**Figure 8:**
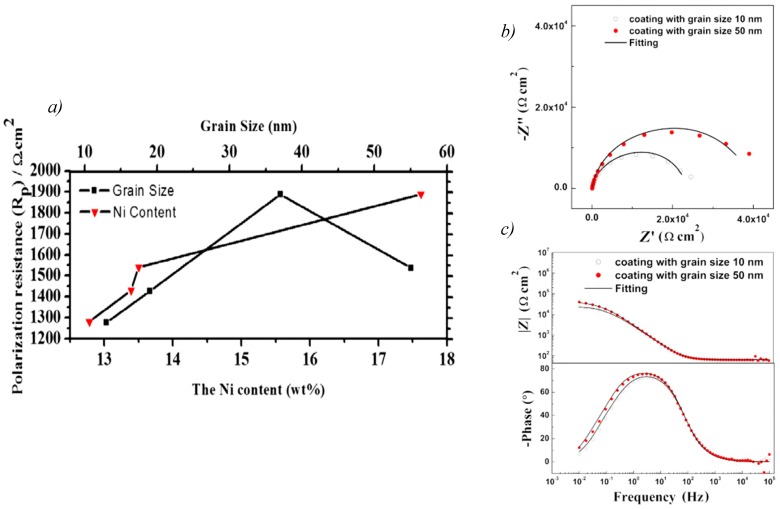
Effect of grain size on the nanoscale. (**a**) Highest polarisation resistance obtained with an intermediate grain size of NC Zn–Ni alloy of different nickel content [48]; (**b**,**c**) Higher impedance and phase values for the higher grain size for coated Q325 steel [59].

The effect of grain size was further studied for different sizes among the nanoscale. A lower or higher nanograin size of the nanomaterial is not necessarily to achieve the same effect in terms of corrosion protection, as a finer nanograin size did not had the best corrosion resistance in all of the research work. Figure 8a shows that nanocrystalline Ni that was electrodeposited had the best corrosion resistance for the lowest grain size among 16-nm, 56-nm, and 250-nm grain sizes [42]. However, the nanocrystalline Ni–Cu alloy (35.8 wt.% Cu) coating with a 12.7-nm grain size had lower corrosion current density in 3 wt.% NaCl solution than the nanocrystalline Ni–Cu alloy (26.0 wt.% Cu) coating with a grain size of 6.6 nm [44].

The same results of better enhancement with the increase of nanograin size was obtained by Meng et al. A bigger value of impedance and of the phase value for Q235 steel coating was observed with grain size of 50 nm compared to that of 10 nm (Figure 8b,c). This indicates that a more stable passive film was formed on the 50-nm coating. In addition, diffraction patterns obtained from the TEM images for the 50-nm coating showed the existence of growth twins. These growth twins form a special boundary with low free energy that significantly lessens the adsorption of aggressive ions through the passive film surface [59]. Also, nanocrystalline Zn–17Ni of an intermediate grain size compared to the other tested Zn–Ni alloys had the best corrosion resistance [48]. This indicates that composition of the nanocoating can dominate the effect of lower grain size. For another kind of alloy, such as the Ni–W alloy, the corrosion resistance of the nanocoating was affected by the acidity of the tested media more than the grain size of that nanocoating [60]. 

#### 2.1.4. Coating Method

**Figure 9:**
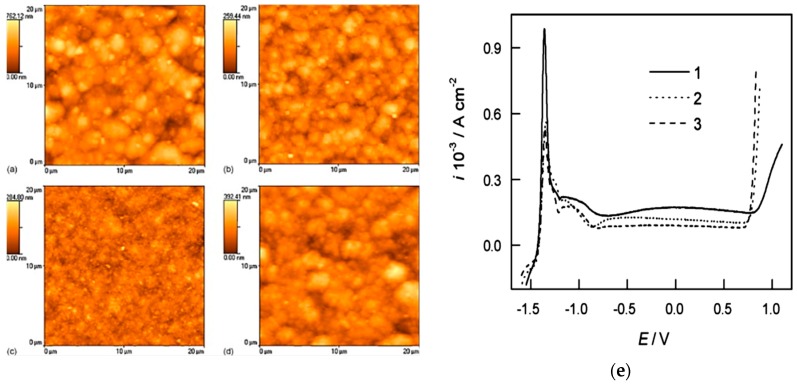
(**a**–**d**) AFM images for zinc deposited under different current densities values (A/cm^2^): (**a**) 0.025 (Direct current plating); (**b**) 0.1; (**c**) 0.3; (**d**) 2; (**e**) Polarization curves for nanocrystalline Zn in 0.1 M NaOH solution: (1) Direct plated (2 and 3) pulse plated of 0.2 A/cm^2^ at a scan rate of 5 and 20 mV/s, respectively [63].

Another factor affecting the corrosion properties of the nanocoating is the coating method. Nanocoating can be obtained with one of the thin film deposition methods, as shown in Figure 3. The coating method defines the surface topography, which greatly influences the nanocoating properties. Nanocoating can be prepared by different methods, and electrodeposition is the most used technique for metallic nanocoating so far [32,42,44,47,48,49]. Electrodeposition is the deposition of a metal or alloy coating over a conducting surface by means of electrolysis from a well-formulated electrolyte, which is known as bath [61]. Dense and pores-free coating is produced with this technique compared to other methods, which would create lots of pores and grain boundaries [44]. Electrodeposition can be performed with either a direct or pulse current. Pulse electrodeposition was shown to enhance deposit properties such as porosity, ductility, hardness, and surface roughness [31,32]. Nanocrystalline zinc deposits that were pulse electrodeposited were more homogenous; they also had a finer-grained surface and a higher number of the lattice imperfections compared to the direct current electrodeposition. This was due to the higher and instantaneous current density during pulse deposition, which increased the nucleation rate and thus led to the formation of finer grains [32,62,63]. Figure 9a–d shows that direct current had a higher size of grains and agglomerated crystals, while an optimal current density of 0.2 A/cm^2^ produced the lowest size of agglomerated particles, which was tested to produce good corrosion behaviour (Figure 9e) [63]. Moreover, nanocrystalline Ni–Cu alloy coating also obtained better corrosion resistance through pulse electrodeposition compared to direct electrodeposition, both in aerated and deaerated environments [44]. 

#### 2.1.5. Additive Type and Concentration

**Figure 10:**
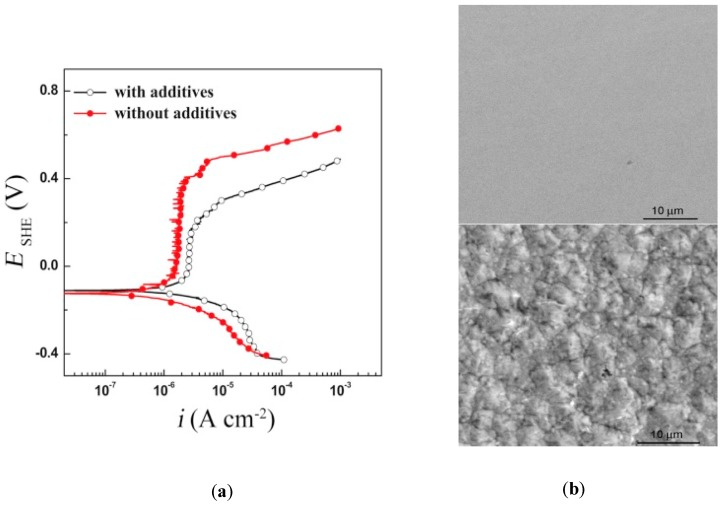
Effect of additive. (**a**) Potentiodynamic polarisation curves of nanocrystalline nickel coatings synthesised with (saccharin and 2-butyne-1,4-diol) and without the additive; (**b**) Surface morphology of nickel coatings synthesised from the bath: (**a**) with additives, (**b**) without additives [59].

The presence of additives in the coating helps form a smoother and shinier surface. Absorbed additive molecules affect the activation energy, the rate of charge transfer, and the mechanism of electrocrystallisation during electrodeposition, as they block the surface and decline the rate of the nucleation of active sites on the surface [64]. Selecting the right additive and its concentration is important to provide an enhancement to the nanocoating material. Nanocrystalline Ni–W alloy coating was electrodeposited on mild steel from a citrate bath containing salicylaldehyde additive. An optimised additive concentration of 100 ppm was revealed to have the best corrosion resistance as it formed a nanocrystalline, uniform, and fine-grained coating. With additive concentrations higher than 100 ppm, the uniformity and corrosion resistance of the nanocoating decreased due to the adsorption and inclusion of the additive in the deposit [47]. The addition of saccharin to an nanocrystalline Ni–Co alloy deposited on carbon steel improved the corrosion and tribocorrosion resistances in 10 wt.% NaOH solutions, as revealed from the polarisation curves in Figure 10a [59]. Such improvement was attributed to the decrease in the grain size and the smoothed surface morphology (Figure 10b), despite the increase in hardness with this addition. On the other hand, the addition of sodium lauryl sulfate to the same coating of an nanocrystalline Ni–Co deposited alloy increased the corrosion resistance, but decreased the tribocorrosion resistance due to its lower hardness [59,65]. 

#### 2.1.6. pH of the Environment

**Figure 11:**
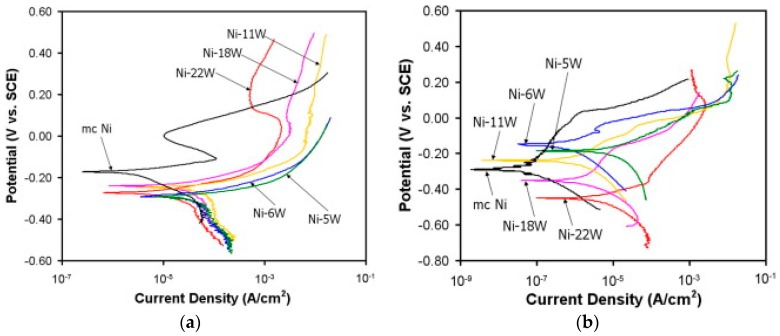
Effect of pH on nanocoating. Potentiodynamic curves of microcrystalline (mc) Ni and various nanocrystalline (nc) Ni–W alloys in 3.5 wt.% NaCl solutions. (**a**) at pH 3; (**b**) at pH 10 [60].

In all of the above-mentioned factors, it was important to specify the test media. The type of test solution affects the pH of the environment, which reflects the capability of the material to resist corrosion attack. Different corrosion current density values were obtained when nanocrystalline cobalt was tested in different solutions of NaOH, HCl, NaCl, and H_2_SO_4_ [43]. Good corrosion resistance was reported even in an alkaline NaOH solution for nanocrystalline zinc deposits (under pH 13.6) [32] and for nanocrystalline iron [53]. Chianpairot et al. reported an increase in the corrosion rates for NC Ni–W alloy in the acidic media (pH 3) more than in the alkaline media (pH 10) of 3.5 wt.% NaCl solutions. Moreover, different pH values of the test media changed the corrosion behaviour of the same alloy. The polarisation curves in Figure 11 show that the corrosion rate increased with the reduction of grain size (increase in W content) in the alkaline media, while the corrosion rate increased with the increase of the grain size (decreased in W content) in the acidic media [60]. 

#### 2.1.7. Surface Morphology of the Nanocoating

Surface morphology is an important factor that is directly related to the corrosion performance of any surface. Actually, it is associated with most of the above-mentioned factors. For example, the coating method defines the surface morphology, grain size, and any preferred orientation of the deposited coating. A nanocrystalline Zn–Ni alloy was electrodeposited with a direct current of different current densities ranging from 0.5 µA/cm^2^ to 8 µA/cm^2^ for the Zn–13Ni to Zn–17Ni alloys, respectively. Surface morphology was rough and nodular, with a cauliflower-like shape. The higher direct current that was used in deposition provided more coherent and compact surfaces, as the size of deposits was larger and uniform with clearer boundaries, which in return caused the best performance for the nanocrystalline Zn–17Ni coating, as revealed from the polarisation curves of the alloys in Figure 6a [48]. The same effect of direct and pulse current deposition appears on the morphology of nanocrystalline Zn, and is shown in Figure 9a [63]. In addition, increasing the tungsten content and reducing the grain size of the Ni–W alloy below 10 nm transformed the alloy from an angular (for MC alloy) to nodular (for nanocrystalline alloy) morphology, which increased the corrosion rate in the alkaline solution, and decreased this rate in acidic media [60]. Another example for the effect of morphology on the corrosion resistance is introducing the additive into the nanocoating. Saccharin added to a nanocrystalline Ni–Co alloy formed a smoother compact surface compared to the pyramidal one without the additive, as presented in Figure 10b. 

Research work related to metallic nanocoatings is summarised in Table 1, along with the most important parameters that define the nanocoatings and their corrosion resistance.

### 2.2. Ceramic Nanocoating

Ceramic nanocoating involves ceramic materials, which are compounds between metallic and non-metallic elements; the most frequently known ceramics are oxides, nitrides, and carbides. Ceramic oxide coatings have a superior benefit over metallic or organic oxides; they provide a better coating, even with a lower thickness, due to their higher hardness and strength [66]. Ceramic nanocoatings have been implemented in many industrial fields due to their attractive thermal and electrical properties, and that they are more resistant to oxidation, corrosion, and wear than metals in high-temperature environments [67]. The most commonly used ceramics are discussed below with the recent research work related to their use as a nanocoating material. All of the work presented in this section that is related to ceramic nanocoating is summarised in Table 2. 

#### 2.2.1. Titanium Oxides Nanocoating

Titanium dioxide is a ceramic material of unique chemical and physical characteristics including self-cleaning [68], ultra violet (UV) protection [69], large refractive index [68], photocatalytic activity [70], and high abrasive and corrosion resistance [71]. Its distinct characteristics have enabled it to be utilised in many promising applications such as photovoltaics, sensing, electrochromics, self-cleaning and self-sterilising construction materials, etc. [69,72]. 

The technique for the surface preparation of the substrate and the deposition method of the nanocoating is an important step that defines the surface’s structure and affects its corrosion properties. Depositing anatase TiO_2_ nanoparticles on the surface of stainless steel by the sol-gel method followed by a hydrothermal post-treatment process increased the corrosion protection of stainless steel when tested in Ringer solution [71]. Following the same preparation technique, three or four layers (464-nm thickness) of TiO_2_ nanoparticles coating the surface of stainless steel samples was revealed to have the best corrosion protection when tested in an NaCl solution. Such uniform coating decreased the corrosion current density by three times, and increased the corrosion resistance by nearly 10 times, compared with bare steel [73]. 

On the other hand, depositing a protective nanocoating through the atomic layer deposition (ALD) method provides the advantage of having a full shield over the surface of the substrate without any pinholes or cracks compared to other depositing methods such as spray pyrolysis, chemical vapour deposition, physical vapour deposition, etc. This was due to the formation of an amorphous phase of deposited nanoparticles that produced dense and conformal films with the precise control of thickness and composition when coated by the ALD method [74]. The same protection can be provided through the ALD method when using a double-layer structure of coatings. A second layer of TiO_2_ over CrN-coated stainless steel blocked cracks and pinholes, and thus shifted corrosion potential (Ecorr) towards more positive values compared to the steel that was only coated with CrN [75]. Although forming multiple layers improves the nanocoating resistance, a successive application of more than five or six layers increases the coatings’ susceptibility for deformation. The residual tension inside multiple films’ thickness in both the upward and lateral deflections is generated, thus inferring mixed conditions on the surface of the substrate. Such stresses generated between the film thicknesses affect the adhesion and defection of the coating, and decrease its corrosion resistance [73]. 

**Figure 12:**
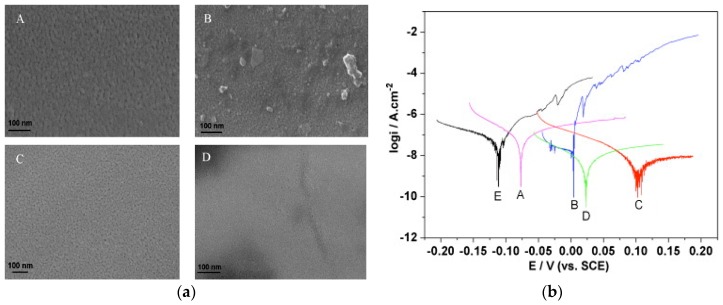
(**a**) SEM images of the different nano-TiO_2_ coated 316L: (A) pure TiO_2_, (B) Cl–TiO_2_, (C) N–TiO_2_, (D) S–TiO_2_; (**b**) Polarisation curves for bare 316L and the different TiO_2_ coatings in 0.5-M NaCl solution: (A) pure TiO_2_/316L coatings; (B) Cl-doped TiO_2_/316L coatings; (C) N-doped TiO_2_/316L coatings; (D) S-doped TiO_2_/316L coatings; (E) bare 316L [77].

The corrosion protection of titania nanocoating was examined to improve coating performance by monitoring the media, size of the particles and thickness of the nanofilm. Decreasing the pH or increasing the NaCl concentration of the test environment, shifted the corrosion potential towards the more cathodic values of the coated steel [73]. A reduction in the size of TiO_2_ nanoparticles achieved an enhancement in the corrosion resistance of nano TiO_2_-coated carbon steel in H_2_SO_4_ solution [76]. Modifying a nano-titania coating by doping it with nitrogen anion improved the coating’s structure and hence corrosion properties of the nanocoating, as it is revealed from the SEM images and potentiodynamic testing in Figure 12a,b, respectively. Doping with nitrogen had the highest corrosion resistance among other doped anions of sulphur and chlorine [77]. 

#### 2.2.2. Alumina Nanocoating

Alumina thin films have unique mechanical properties and corrosion resistance; as a result, they have been implemented in many industrial fields such as surface passivation [78], gas diffusion barriers [79], anti-reflection layers [80], etc. 

The preparation method of alumina nanocoating was investigated for its effect on the corrosion properties of nanocoating. Ruhi et al. reported a better corrosion resistance of 9Cr-1Mo ferric steel coated with nanostructured sol-gel alumina nanocoating compared to the uncoated steel in an NaCl solution [81]. A plasma-enhanced ALD method gave better film density (less porosity) than the thermal ALD method due to its higher reactivity, which improved the nucleation properties of the films [82].

**Figure 13:**
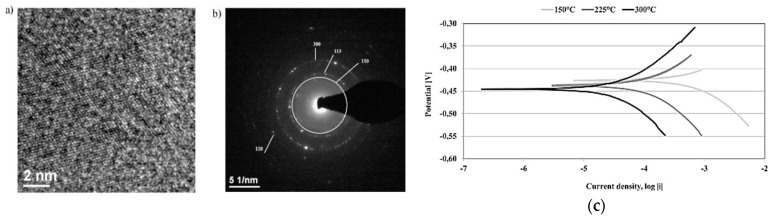
(**a**,**b**) High-resolution (HRTEM) micrograph of the Al_2_O_3_ layer deposited at 300 °C with corresponding SAED pattern; (**c**) Potentiodynamic polarisation curves of the Al_2_O_3_ coating of different deposition temperatures tested in 1-M HCl solution [83].

The ALD method has the advantage of obtaining a controlled, smooth, and equally-coated surface. The deposition temperature with this method can be achieved at temperatures from room temperature to 500 °C. High-resolution TEM (HRTEM) images and polarisation tests showed that the best quality films in terms of density and purity are obtained when prepared under higher temperatures of 300 °C, as shown in Figure 13a–c, respectively [83]. Alumina nanocoating over 316L stainless steel showed a better corrosion performance for coating prepared with ALD at 250 °C compared to the same materials prepared at 160 °C. Higher temperatures reduced the porosity and hence increased the sealing performance of that coating [84]. However, some carbon steel cannot maintain processing under high temperatures, so deposition on carbon steel should be done at lower or at room temperature so as not to affect its structure. The nanocoating of alumina was deposited on 100Cr6 carbon steel with the ALD method at 160 °C. It was revealed that the thickness of the coating needs to be higher than 10 nm to prevent corrosion. Such thickness is required to seal defects resulting from the initial stages of layer growth [85]. Achieving a good level of sealing at room temperatures was performed with the plasma-assisted ALD (PA-ALD) method. This technique purges oxygen plasma radicals in the process, which enables deposition at room temperatures and without requiring long purging times [86]. An excellent adhesion and the corrosion protection of the film was reported with PA-ALD over two kinds of substrates: aluminium alloy and 100Cr6 steel [82]. In addition, better corrosion performance was achieved with a higher thickness of the coating over a 316L stainless steel substrate [84]. It was revealed that 50-nm nanocoating blocks the metastable and stable pitting in the passivation region, and reduces the corrosion rate compared to a 10-nm nanocoating [84]. However, a 50-nm alumina coating prepared with the ALD method at 160 °C failed to protect the carbon steel substrate in 0.2-M NaCl solution. This was an unexpected result, as Al_2_O_3_ is stable under neutral conditions, according to the Pourbaix diagram. ALD-coated substrate corroded under higher temperatures due to the type of substrate, since carbon steel caused a reduction of oxygen and introduced OH– ions, which increased the pH and initiated the dissolution of the oxide layer [87]. 

**Figure 14:**
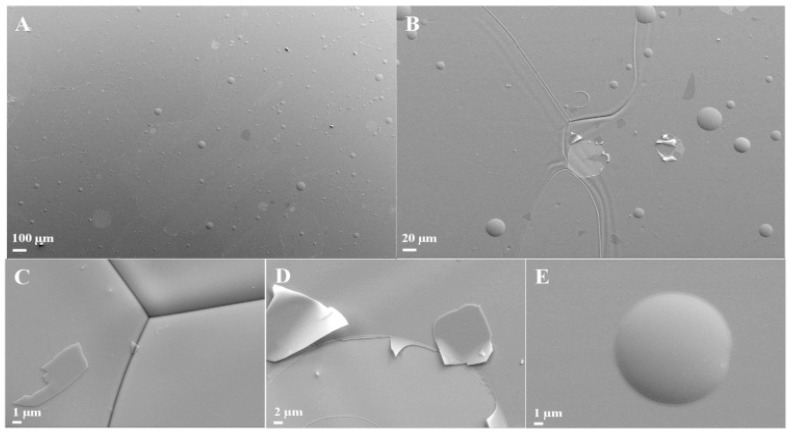
FEG-SEM images for the pristine 50-nm atomic layer deposition (ALD) alumina-coated sample prepared on the annealed copper substrate [89].

The pre-treatment process affects the structure and the topography of the substrate, and can be performed to enhance the properties of the substrate, but it might have an adverse effect as well. The pre-treatment of carbon steel with H_2_-Ar plasma, which has 50 nm of Al_2_O_3_ deposited by either the thermal or plasma-enhanced ALD method, will increase the corrosion resistance as well. Such treatment increases the adhesion and reduces the porosity of the coated surface [88]. Pre-annealing of the substrate before coating removes all of the heterogeneities and lowers the surface roughness. Following such a procedure with copper substrate and then coating it with 10-nm alumina films by the ALD method increased the corrosion protection, as was observed from the potentiodynamic and EIS testing. This protection decreased as the thickness increased, which was due to the weak adherence that resulted in local detachments of the deposited alumina films. Hence, increasing the film thickness of the ALD-deposited alumina from 10 nm to 50 nm reduced the corrosion protection of the pre-annealed coated copper, as indicated by the flasks of missing pieces that appear in the SEM images of Figure 14 [89]. 

#### 2.2.3. Tantalum Oxide Nanocoating

Pentoxide (Ta_2_O_5_) is a refractory metal that exhibits attractive physical, structural, optical, and electrical properties such as high dielectric strength [90], high hardness [91], and high chemical attack resistance under severe conditions [92]. It is used in many applications, from microelectronics to the chemical and biomedical industries such as capacitors, biological and chemical sensor layers, anti-reflection coatings, and optical waveguides [90,93].

**Figure 15:**
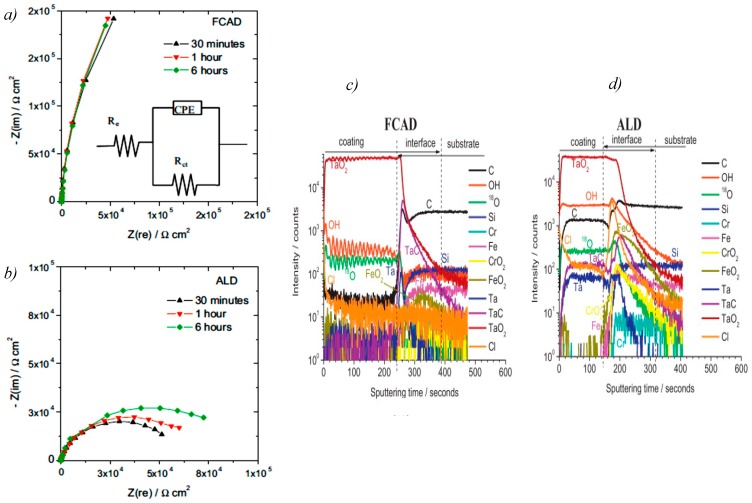
Nyquist plots for the filtered cathodic arc deposition (FCAD) (**a**) and ALD (**b**) coated 100Cr6 substrate during immersion in neutral 0.2-M NaCl solution. Time-of-flight secondary ions mass spectrometry (ToF-SIMS) negative ions depth profiles for the 50-nm tantalum oxide layer prepared by FCAD (**c**) and ALD (**d**) on the 100Cr6 substrate [95].

Research works related to pentoxide nanocoating showed enhancement in corrosion properties. Coated Ti-6Al-4V alloy with β-Ta_2_O_5_ had a stable passive oxide film on its surface when tested in NaCl solution for corrosion properties. Coated alloy showed higher open corrosion potential and lower corrosion current density compared to bare Ti–6Al–4V alloy [94]. Carbon steel coated with 50 nm of tantalum oxide also did not exhibit any film dissolution in the acidic or neutral media of an 0.2-M NaCl solution [92]. Diaz et al. investigated the effect of coating method on the performance of tantalum oxide nanocoating over a 100Cr6 steel substrate. The substrate was coated by the filtered cathodic arc deposition (FCAD) method and by atomic layer deposition (ALD). The corrosion characteristics for the steel coated by FCAD were shown to be better than those of the samples coated by ALD, as indicated by the higher values of charge transfer resistance for the FCAD samples. The Nyquist plot for FCAD (A) in Figure 15a,b showed a bigger diameter of the semi-circles at a high-frequency range for the FCAD plot, which points out the larger global impedance for FCAD compared to that for ALD samples. This was explained by the presence of carbonaceous contaminations on the surface of the ALD film; this affected its sealing, as it was identified from the time-of-flight secondary ions mass spectrometry (ToF-SIMS) in Figure 15c,d. On the other hand, the pre-etching stage of the FCAD method nearly removed all traces of iron and chromium oxides from its surface, giving it lower porosity. Such surface refinement achieved a reduction in corrosion rate by a factor of four for the tantalum oxide film prepared by FCAD compared to that of the ALD-coated surface [95]. Moreover, tantalum nitrate (T_2_N) is another ceramic that exhibited a decrease in the corrosion resistance with the increase in acidity and temperature [29]. 

#### 2.2.4. Zirconia Nanocoating

Zirconia (ZrO_2_) has many applications in different fields due to its promising physical and chemical properties such as low friction coefficient, high melting point, high chemical stability, high refractive index, and dielectric constant [96,97,98]. Zirconium thin film is used in many industrial applications as thermal barrier coatings, for the corrosion protection of metals, optic devices, magnetic storage media, catalyst support, etc. [99,100,101]. It is widely implemented as a coating material due to its high corrosion resistance, long wear life, and high-temperature resistance [102]. Thin films of zirconia can be deposited by various methods, such as sol-gel [27,103], hydrothermal process [101], thermal spraying [104], electrodeposition [50], and ion beam-induced chemical vapour deposition [105]. However, sol-gel showed to be the most favourable method, since a homogenous barrier film can be produced on large and complex surfaces at relatively low cost [106]. In addition, a higher degree of purity and easier control of stoichiometry can be achieved for the oxide layer produced with the sol-gel method [103]. 

**Figure 16:**
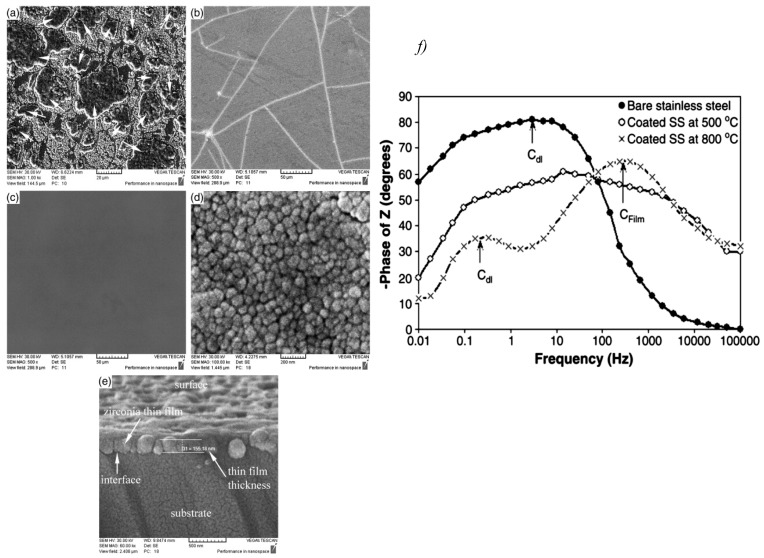
(**a**–**e**) SE-SEM images of: (**a**) chromium oxide on the bare substrate; (**b**) cracked ZrO_2_ thin film at 800 °C; (**c**) suitable nanostructure films at 500 °C (**d**) top view of thin film at 800 °C and (**e**) a 7° tilted cross section of the film at 800 °C with a thickness of 155 nm on the substrate. (**f**) Bode diagram of bare stainless steel and ZrO_2_-coated samples tested in 1-M H_2_SO_4_ solution at 80 °C. Coated samples were heat treated at 500 and at 800 °C [109].

Zirconia thin films prepared with the sol-gel method formed a barrier to the corrosive media and showed an enhanced corrosion rate of 316L stainless steel in both acidic and neutral media [103]. Such thin films enhanced the corrosion protection by decreasing the porosity, and hence decreasing the penetration of the aggressive ions into the substrate surface [107]. The same barrier protection effect was maintained when Li et al. performed a heat treatment on zirconia-coated magnesium alloy up to 360 °C [108]. Other research work [109] examined the corrosion behaviour of zirconia nanocoating on 316L stainless steel at temperatures up to 900 °C. SEM images shown in Figure 16a–e revealed that calcination treatment at temperatures higher than 500 °C formed pinholes and pores and decreased barrier properties. In addition, one time constant was observed at low frequencies for the zirconia-nanocoated film treated at 500 °C, indicating that the corrosion reactions were controlled by the double-layer capacitance, as shown in the Bode plots of Figure 16f. On the other hand, two time constants were observed for the film treated at 800 °C; the first one was due to the faulty structure and porosity of the film, and the second one was attributed to the substrate corrosion. In agreement with EIS testing, SEM images showed crack-free thin films for the surfaces treated at a calcination temperature of 500 °C, which also had the best corrosion performance [109]. The same results were obtained for electrodeposited and then thermally treated zirconia nanocoating in Hank’s solution [50]. 

Other factors affecting the barrier properties of the nanocoating include the adhesion properties and layer thickness of the coating. Zirconia coating is a promising replacement coating for chromium. It requires three layers dipping of zirconia nanocoating on an aluminium alloy, and then heat treatment at 250 °C to achieve the same corrosion protection as chromatid coating [27]. Despite the good corrosion and adhesion properties it showed in another previous work [107], this coating had less self-healing properties compared to chromatid coating [27]. 

The method of nanocoating preparation plays a role in identifying the properties of the coating. Hydrothermal deposition [101], thermal spraying [104] and ion beam-induced chemical vapour deposition (IBICVD) [105] achieved better corrosion properties compared to a bare substrate. Hydrothermal deposition is widely recognised in industrial applications due to its simplicity. Pre-oxidising the substrate’s surface with α-Fe_2_O_3_ film gave better adhesion and compactness of the hydrothermally coated substrate [101]. The same results of enhancing compactness was achieved with the IBICVD method, even at a low layer thickness of 10 nm [105]. The thermal spraying of zirconia achieved a corrosion enhancement of a titanium and a titanium alloy substrate at low cost [104]. On the other hand, spray pyrolysis was used to cover large areas with the thin films at low cost, but it is challenging to get dense and homogeneous thin films out of this process [110]. Hence, choosing the right method is important to achieve a continuous, compact, and dense zirconia layer.

**Table 2:** Summary of some corrosion parameters of ceramic nanocoatings.

Nanomaterial Coating	Coating Thickness	Substrate	Electrolyte	Corrosion Resistance	Tested Conditions	Ecorr (V vs. SCE)	Icorr (µA/cm^2^)	Ref.
**Titanium Oxides**								
TiO_2_ anatase NP (φ 15–18 nm)	375 nm	316L Stainless Steel	Ringer solution	Conformal thin layers of TiO_2_ formed an entire shield over the substrate	-	No values provided. Only graph		[71]
TiO_2_ NP (φ 40 nm, pore size 5–8 nm)	375 nm, 464 nm, 550 nm	316L Stainless Steel	0.5 mol/L NaCl solution	Best for 464-nm coating thickness. Increasing NaCl concentration or decreasing pH increased corrosion	375-nm TiO_2_ thickness	−0.011	0.00897	[73]
					464-nm thickness	0.027	0.000105	
					550-nm thickness	−0.117	0.783	
Amorphous TiO_2_ NP films	50 nm	316L Stainless Steel	3 wt.% NaCl solution	Conformal and dense thin layers of TiO_2_ formed an entire shield over the substrate	Bare stainless steel	−0.96	0.7	[74]
					TiO_2_ coated stainless steel	−0.63	0.063	
TiO_2_ thin films	370 nm	316L Stainless Steel	0.5-M NaCl Solution	Best with the N-modified TiO_2_ surface	-	No values provided. Only graph		[77]
Amorphous TiO_2_ NP layer over CrN coated SS	90 nm	316L Stainless Steel	3 wt.% NaCl solution	Conformal thin layers of TiO_2_ formed an entire shield over the coated substrate	Bare stainless steel	−0.95	0.0026	[75]
					CrN single layer over stainless steel	−0.74	0.0019	
					CrN/TiO_2_ over stainless steel	−0.49	0.00031	
**Tantalum Oxides**								
equiaxed ß Ta_2_O_5_ (avg. grain size ~20 nm)	25 µm	Ti-6Al-4V	3.5 wt.% NaCl solution	Enhanced for coated samples	Uncoated Ti-6Al-4V	−0.54	0.501	[94]
					Ta_2_O_5_ nanocoated Ti-6Al-4V	−0.26	0.117	
T_2_N (grain size 13 nm)	-	Ti-6Al-4V	0.5 M H_2_SO_4_ solution	Increasing the acidity and temperature decreases the corrosion resistance	-	No potentiodynamic test done		[29]
Thin films of tantalum oxide (Ta_2_O_5_)	10, 50 nm	Carbon steel	0.2-M NaCl solution	Icorr decreases with increasing grain size (best for 50 nm). Ta–O nanocoating better than Cr–O	Ta–O (10 nm)	−0.714	0.169	[92]
					Cr–O (10 nm)	−0.753	0.428	
					Ta–O (50 nm)	−0.671	0.0348	
					Cr–O (50 nm)	−0.693	0.208	
Thin films of tantalum oxide (Ta_2_O_5_)	50 nm	Low alloy steel	0.2-M NaCl solution	Corrosion rate of coated steel by FCAD is four times less than that of the ALD	ALD at pH 7	−0.79	0.093	[95]
					FCAD at pH 7	−0.67	0.039	
**Aluminium Oxides**								
Thin films of Al_2_O_3_ deposited	50 nm	carbon steel	0.2-M NaCl solution	Failed to protect the steel	-	No potentiodynamic test done		[87]
Al_2_O_3_ (from 8–12 nm nanoparticles)	_	9Cr-1Mo steel	NaCl solution	Enhanced at both concentration compared to bare substrate, but the coating was susceptible to pitting under 200 ppm of Cl-conc.	-	No values provided. Only graph		[81]
Al_2_O_3_ thin film deposited	10 nm, 50 nm, and 100 nm	100Cr6 carbon steel	Deaerated 0.2-M NaCl solution	Corrosion rate decreased by one, two, and four orders of magnitude for the coating thicknesses of 10 nm, 50 nm, and 100 nm, respectively	-	No values provided. Only graph		[85]
Al_2_O_3_ thin film deposited	50 nm	100Cr6 carbon steel	Deaerated 0.2 M–NaCl solution	Enhanced for thermally ALD more than for plasma ALD one.	-	No values provided. Only graph		[88]
Al_2_O_3_ thin film deposited	10–50 nm	100Cr6 carbon steel, Al2024-T3 alloy	Deaerated NaCl solution	Best corrosion for steel and Al alloy was at 150 °C and 50 °C, respectively. Better with PEALD than thermal ALD.	-	No potentiodynamic test done		[82]
Al_2_O_3_ thin film deposited	200 nm	X40CrMoV5-1 steel	1-M HCl solution	Best with 300 °C deposition temperature	ALD at 150 °C	−0.43	670	[83]
					ALD at 225 °C	−0.447	190	
					ALD at 300 °C	−0.456	50	
Al_2_O_3_ thin film deposited	10, 20, 50 nm	Copper	Deaerated 0.5-M NaCl solution	10 nm better than 50 nm	10-nm thickness of Al_2_O_3_	−0.356	0.15	[89]
					20-nm thickness of Al_2_O_3_	−0.336	1.52	
					50-nm thickness of Al_2_O_3_	−0.308	2.71	
Al_2_O_3_ and Ta_2_O_5_ thin film deposited	5–50 nm	316L stainless steel	Deaerated 0.8-M NaCl solution	Better with higher thickness and higher deposition temperature. Al_2_O_3_ nanocoating better than Ta_2_O_5_.	-	No values provided. Only graph		[84]
**Zirconium Oxides**								
Thin films of ZrO_2_	10, 35, 100 nm	Brass	Borate buffer (BB) and BB + 0.5-M NaCl solution	All showed a protective effect of the nanocoating.	-	No potentiodynamic test done		[105]
Thin films of ZrO_2_	50–350 nm	Pre-oxidised 304L stainless steel	0.1-M Na_2_PO_4_ solution	Best when oxidising the surface before coating	Uncoated substrate that is pre-oxidised with water and oxygen, and then with Fe_2_O_3_	−0.2475	1.972	[101]
					Zirconia-coated substrate that is pre-oxidised with water and oxygen, and then with Fe_2_O_3_	−0.1922	0.104	
Thin films of ZrO_2_	70–180 nm	Aluminum alloy AA6060	Diluted Harrison solution (0.05 wt.% NaCl + 0.35 wt.% (NH_4_)_2_SO_4_)	Three dips of zirconia coating gave the same barrier properties as chromatised substrate	-	No values provided. Only graph		[27]
Thin films of ZrO_2_	155 nm	316L stainless steel	1-M H_2_SO_4_ solution	Best with heat treatment temperature of 500 °C	Coated at 300 °C	−0.1814	3.11	[109]
					Coated SS at 500 °C	−0.152	0.65	
					Coated SS at 600 °C	−0.1673	2.88	
Thin films of ZrO_2_	-	AZ91D magnesium alloy	3.5% NaCl solution	Best with treatment temperature of 360 °C	Zirconia-coated at 120 °C	−1.5651	1.98	[108]
					Zirconia-coated at 240 °C	−1.5468	1.43	
					Zirconia-coated at 360 °C	−1.5155	0.526	
Thin films of ZrO_2_	0.4–0.6 µm	316L stainless steel	Deaerated H_2_SO_4_ and in 3% NaCl solutions	Presented barrier properties in both acidic and neutral solutions	-	No values provided. Only graph		[103]
Thin films of ZrO_2_	0.5–0.8 µm	316L stainless steel	Hank solution	Better with samples treated at 400 °C more than 650 °C	-	No values provided. Only graph		[50]
Thin films of ZrO_2_	500 nm	Alumina–silica refractory material	Molten borosilicate glass at 1400 °C for 162 h.	For zirconia-coated refractory, porosity and corrosion loss decreased by 18% and 16%, respectively.	-	No potentiodynamic test done		[107]
Thin films of ZrO_2_	150 µm	Cp–Ti and Ti–13Nb–13Zr alloy	Hank solution	Almost same corrosion enhancement on the two substrate by coating with zirconia.	Al_2_O_3_-13 wt.% TiO_2_ coating on cp-Ti	0.306	1.77	[104]
					ZrO_2_ coating on Ti–13Nb–13Zr	0.516	3.79	
					ZrO_2_ on cp–Ti substrate	0.411	3.02	

#### 2.2.5. Other Ceramic Nanocoatings

Coating with graphene layers showed to have corrosion resistance that was better than the organic ones, which are more than five times thicker. When multi-layer graphene films deposited by chemical vapour deposition (CVD) over copper films (25-µm thickness), the corrosion rates of copper in an aerated Na_2_SO_4_ solution were seven times slower than that of bare copper. Similarly, a multi-layer graphene coating on the surface of nickel films revealed a corrosion rate in the same electrolyte solution that was 20 times slower than that for bare nickel [111].

Nanosized hydroxyapatite (n-HA) has been deposited on orthopaedic implant material (titanium alloy; Ti6Al4V) and characterised for its corrosion resistance in Hank solution. During corrosion testing, for n-HA coated titanium alloys, the initial open circuit potential (OCP) continued to increase until the protective air–oxide film reached its protective capacity. This shows that n-HA coated titanium alloys are robust towards corrosion resistance [112].

### 2.3. Nanocomposite Coating

Nanocomposite coating is a material composed of at least two immiscible phases, which are separated by an interface region. The main component in nanocomposite coating is the matrix, in which the filler is dispersed [113]. The matrix is the dominant part of the composite, which is usually the material that possesses properties to be enhanced. It can be metallic, ceramic, or polymer, with a dimension larger than nanoscale. Fillers are nanomaterials that can be in the 0D, 1D, and 2D nanoscale, and the nanocomposite coating is categorised according to that dimension of the filler. Fillers can be used as nanoparticles (0D); nanotubes, nanowires, or nanorods (1D); or nanoplatelets, nanosheets, or nanofilms (2D). A 2D nanocomposite coating can have a laminate or sandwich form. Laminate nanocomposite is composed of thin layers at nanolevel thickness of two or more different class materials [114]. Meanwhile, the sandwich nanocomposite is a large-scale layer (thickness > 100 nm) deposited on both surfaces of a nanoscale layer [115]. The main purpose of composing two different materials in one coating is to have a new nanocomposite material with distinctive characteristics and superior properties compared to each material individually. Nanocomposite coating achieves an improvement in the mechanical properties, in addition to increasing corrosion resistance. 

Common nanocomposites that are used for coating are matrix-reinforced. Polymer and metallic matrices nanocomposite coatings will be discussed in the following sections. Their corrosion behaviour will be the major concern, as well as how the enhancement in different properties will affect it. In the polymer nanocomposite field, a conductive polymer nanocomposite where the host matrix used is a conductive polymer will be discussed for its importance and huge potentials in different applications. In addition, a recent type of polymer nanocomposite coating, which is waterborne, will be studied for its corrosion behaviour. In the metallic/ceramic nanocomposite section, an electroless Ni nanocomposite will be briefly discussed due to its improved characteristics over the conventional electrodeposited techniques. At the end, Table 3 summarises the reviewed polymer nanocomposite coating papers.

#### 2.3.1. Polymeric Host Matrix of Nanocomposite Coatings

Polymer nanocomposite coatings have evoked a great deal of interest in the corrosion protection applications due to the extraordinary properties that they can offer. With polymers used as host matrices in various composite films, nanomaterials are incorporated in the polymer matrix as a filler or pigment. When fillers are embedded into a polymer, the resulted hybrid organic–inorganic material is known as a polymer nanocomposite [116,117]. 

Usually, nanofillers are incorporated in the polymer matrix in order to increase the stiffness, strength, conductivity, and thermal resistance; they also reduce the thermal expansion, permeability, solvent attack, flammability, and fouling, and retain elongation, transparency, density, processability, cost, and chemical resistance [118,119,120,121]. Polymers are very noble towards corrosion, with poor scratch and wear resistance. The incorporation of ceramic nanofillers into a polymer matrix improves the hydrophilic, anti-wear, and self-healing properties, which will result in increasing the corrosion resistance. When the ceramic alumina or silica nanoparticles are incorporated in paints, they improve the anti-scratch property; also, when clay is incorporated into the polymer matrix, the porosity and the diffusion paths of the resulting nanocomposite are reduced due to the increase in the gas and water barrier properties [122]. 

Processing polymer nanocomposites is critical. Depending on the synthesis process, we may end up with either a perfect dispersion of nanofiller in the polymer matrix or with an agglomerated nanocomposite. The perfect dispersion and distribution of nanofillers in the matrix reflect the best possible enhancement in the properties. Several chemical processes are used to mix a polymer matrix with solid nanoparticles such as in situ polymerisation, emulsion polymerisation, solution intercalation, and melt intercalation [123,124,125,126]. Both in situ polymerisation and solution mixing are considered to have the best polymer nanocomposite in terms of nanoparticles’ dispersion and distribution.

**Figure 17:**
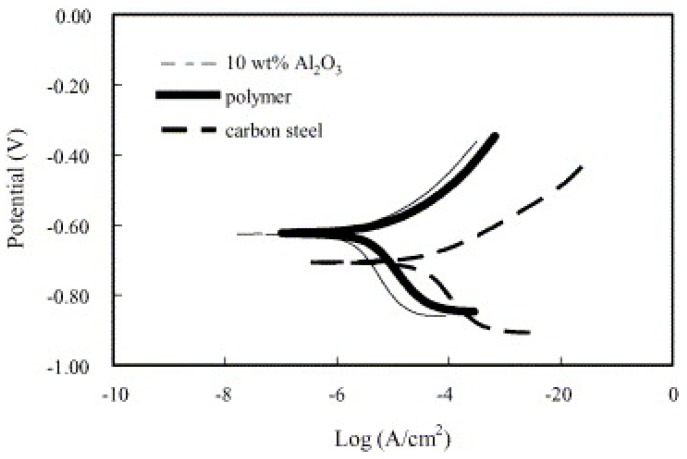
Potentiodynamic curves in 3 wt.% NaCl solution for bare carbon steel compared with that for carbon steel coated with polymer and coated with 10 wt.% Al_2_O_3_ filler added to the polymer coating [128].

In the literature for polymer nanocomposites that coat the surface of stainless steel, different fillers are used to incorporate with polymers. MWCNT [127], Al_2_O_3_ [128], graphene oxide (GO) [120], ZrO [129], and SiO_2_ [130] were added to epoxy and vinyl chloride/vinyl acetate copolymer (VYHH), Xylan 1810/D1864, chitosan (CS), epoxy resins (EP), and fluoropolymer matrices, respectively, for corrosion protection purposes. The addition of 0.1 wt.% MWCNT in both epoxy and VYHH coating showed improvement in the mechanical strength and corrosion protection in NaCl solution. This improvement can reflect the enhancement of the cohesive and adhesive properties of the nanostructure. This enhancement can be attributed to the proper dispersion of MWCNT in the matrix [127]. When Al_2_O_3_ nanoparticles were blended into the polymer, the mechanical properties were strengthened while sustaining the corrosion resistance of the polymer itself. The potentiodynamic test results in Figure 17 show a decrease in the corrosion current density of the nanocomposite coating with 10 wt.% filler compared to only the polymer coating and the uncoated carbon steel in the NaCl solution. Also, both scratch and wear resistance were enhanced with the increase of nanoparticle weight percent in the matrix. The scratch resistance was two times higher than that of the polymer [128]. 

**Figure 18:**
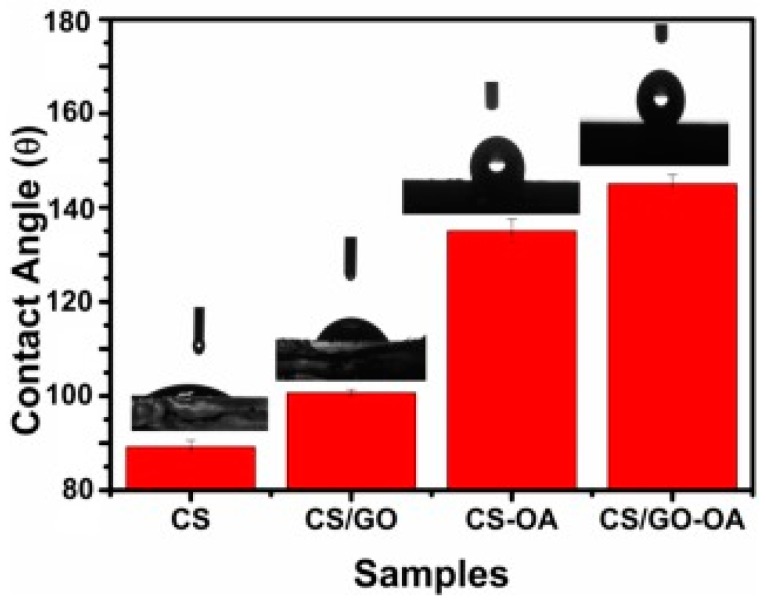
Water contact angles for chitosan (CS), CS/graphene oxide (GO), CS–oleic acid (OA) and oleic acid-grafted chitosan/graphene oxide (CS/GO-OA) [120].

Another property that can be enhanced by the addition of nanofillers is hydrophobicity, which can return an enhancement in corrosion resistance. The introduction of GA and oleic acid (OA) to the chitosan to produce the nanocomposite coating of CS/GO-OA (oleic acid-grafted chitosan/graphene oxide), improved the corrosion protection over carbon steel in an NaCl solution. The large alkyl group of the OA resulted in the higher hydrophobicity of the coating’s surface, as it is revealed by the higher surface contact angle with the water that is shown in Figure 18. In addition, the interaction of the functional groups between the chitosan and the OA chains provided an efficient barrier and prevented ionic transportation from the NaCl solution into the surface of the coating. This caused a reduction in the hydrophilicity, oxygen permeability, and transportation of ions through the film, which in sequence increased the corrosion resistance 100-fold [120].

**Figure 19:**
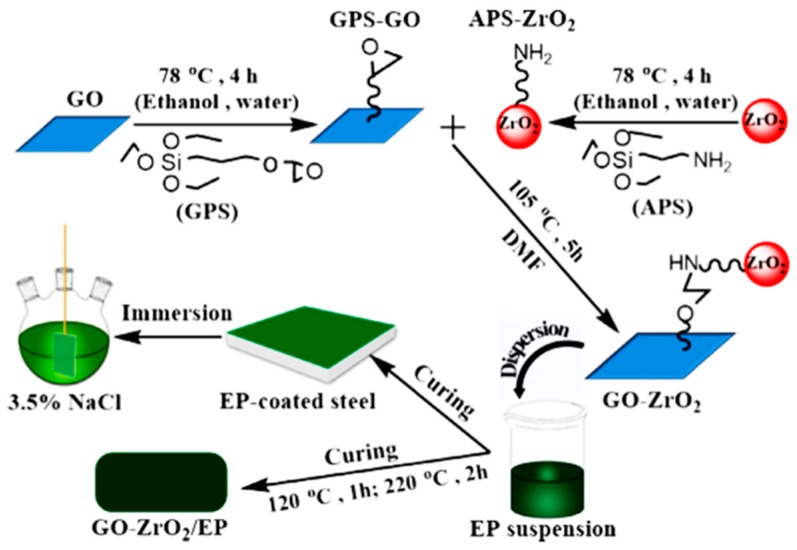
Schematic of preparation of GO–ZrO_2_ and the corresponding hybrid coatings [129].

A hybrid nanocomposite coating of graphene oxide–zirconia dioxide/epoxy (GO–ZrO_2_/EP) was prepared and then coated over a steel sheet, as shown in Figure 19 [129]. Studying the distribution and exfoliation of GO–ZrO_2_ in the EP using FE-SEM images showed the absence of clusters’ aggregation. In addition, the results showed an improvement in the adhesion strength of the interface between the coating and the metal substrate when GO and ZrO_2_ were incorporated with the EP. When tested for corrosion resistance in NaCl solution using EIS, the hybrid coating (GO–ZrO_2_/EP) showed the highest resistance compared to EP, GO/EP, and ZrO_2_/EP coatings. The combination of the GO–ZrO_2_ provided a physical barrier to obstruct electrolyte permeation, since they have a 2D sheet structure, high aspect ratio, and uniform dispersion and exfoliation within the EP matrix [131,132,133]. 

**Figure 20:**
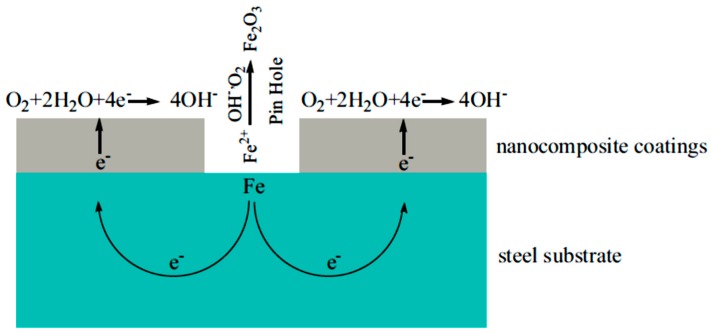
Schematic of anti-corrosion mechanism for the nanocomposite coating [130].

Incorporating nanoparticles on the coating’s matrix has a big role in corrosion retardation. Treated silica nanoparticles were introduced into the fluoropolymer matrix in order to improve the dispersibility in the polymeric coating. It was identified that the corrosion resistance has improved up to the addition of 5% concentration of the SiO_2_; after that, loading corrosion starts. SEM images showed that after that concentration, the nanoparticles start to agglomerate, and the bond strength of the coating–substrate starts to decrease [130]. Nanocomposite coatings can reduce corrosion by more than one act. The barrier effect by the formation of passive film on the surface of the nanocomposite coating is one effect, in addition to the fine distribution of electrical conductivity inside the polymeric matrix, which enables corrosion protection. Furthermore, oxygen reduction on the surface of the polymer can provide low overpotential areas and reduce corrosion reactions on the surface. Figure 20 shows a schematic diagram of a proposed corrosion mechanism on the surface of a nanocoating. 

##### Conductive Polymer Matrix Nanocomposite Coating

Conductive polymers or, more precisely, intrinsically conducting polymers, have received a great interest in recent years due to their electrochemical properties [134]. They have been used as host matrices in various composite films. Common conductive polymers are polyaniline (PANI), polythiophene, and polypyrrole. It was revealed that the presence of these conductive polymers enhances the corrosion resistance of the nanocomposite coating [135]. 

Polymer coatings modified with nanocomposite have been tested for their anti-corrosion properties as coatings for steel protection. The PANI nanocomposite tends to enhance the resistivity of the coating with its redox behaviour and self-healing effect when it is threatened to be destroyed due to scratch or scribble [136,137,138]. Moreover, a modified PANI coating with nanoparticles of TiO_2_, ZnO, CaCO_3_, and graphene revealed superior corrosion resistance [121,136,137,138,139]. A PANI–TiO_2_ nanocomposite coating showed more than 100 times’ improvement in the corrosion resistance, especially for PANI prepared with 4.18 wt.% nano TiO_2_ [136]. The same finding was obtained for a PANI hybrid coating containing nanoparticles of ZnO in a polyvinyl acetate (PVAC) matrix [137]. This exceptional improvement has been associated with the presence of nanoparticles, since they tend to increase the diffusion resistance, prevent charge transportation, and increase the surface area available for the liberation of the dopant [136,137]. The dopant anion here acts similar to a corrosion inhibitor, and helps passivate the steel surfaces, even if the coating is breached. When breached is occurred, the anodic corrosion reaction causes a reduction of the polymer composite film (which acts similar to a cathode to the stainless steel plate) instead of the steel surface, as it exists in the doped acidified state. When PANI was integrated with water-repellent CaCo_3_ into alkyd resin, the corrosion rate was found to decrease appreciably with the increase of the PANI–CaCO_3_ (PAC) nanocomposite loading in alkyd resin, together with the increase in the impact strength of the nanocomposite polymer (PANI) [121]. The ceramic nanocomposite coating of TiO_2_/Graphene oxide (TiO_2_/GO) showed better stability in seawater than the polymeric nanocomposite coating of polyvinyl alcohol/polyaniline/few-layers graphene (PVA/PANI/FLG) when both were used to coat cast-iron pipelines. Such a better corrosion performance was due to the lower population of observed pores in the ceramic nanocoating. In addition, polymeric nanocoating had higher capacitance and was able to store higher charge, which allowed faster degradation [140].

Another nanocomposite examined for corrosion properties was highly crystalline graphene integrated polyaniline (PaniGn) nanostructured composites over mild steel; it was measured for different concentrations of graphene. The results showed a decline in corrosion current of up to four orders of magnitude in HCl solution, where 1.92 wt.% graphene loading showed the best corrosion protection. It was suggested that the coating provided a physical barrier to the corrosive environment and imparted non-wetting characteristics [138]. Furthermore, the PaniGn nanocomposite coating showed a reduction in the corrosion current over copper. This reduction was due to the dense and compact barrier layer ofthe polyaniline coating reinforced with graphene [139]. In addition, the PaniGn nanocomposite coating improved the hydrophobic surface and increased the interaction energies between the graphene and polyaniline [141]. 

##### Waterborne Polymer Nanocomposite Coating

Current paint formulations contain volatile organic compounds (VOCs) as plasticisers to facilitate polymer diffusion, reduce ductility, and increase the flexibility of the paint. However, such technology showed a negative environmental impact [142]. One of the potential alternatives for VOCs to produce solvent-borne polymer coatings is a waterborne polymer coating, where water acts as a solvent instead of the VOCs [143]. Compared to the health hazards and toxicity problems caused by VOCs [144], waterborne polymer coatings have advantageous properties, including eco-friendliness, low viscosity, easy cleaning, and non-toxicity [142]. Waterborne coating uses water as a solvent to disperse a resin.

Polymer-based waterborne coating integrated with nanoparticles such as Fe_3_O_4_, Fe_2_O_3_, and ZnO were studied for their corrosion behaviour [142,145,146]. An exciting technique of waterborne coating is water-based alkyds coating, which is considered to be one of the cheapest VOC-compliant coatings, and can be applied as spray or dip application. It takes a longer time to dry than conventional solvent-borne coating, but the result is the same. Similar results were found when nano-ZnO and nano-Fe_2_O_3_ were incorporated into an alkyd-based waterborne coating system in different concentrations [145,146]. It was found that the addition of a small concentration of nanoparticles can reduce the corrosion rate, while achieving improvement in UV resistance, scratch resistance, and abrasion resistance of the coating as compared to the neat coating system [145,146]. Ferrite (Fe_3_O_4_) dispersed in waterborne epoxy acrylate-butylated melamine formaldehyde (EpAc-BMF) coatings showed a nobler reaction towards corrosion compared to a neat steel substrate [142]. A physical barrier layer was formed by the epoxy coating to control the access of aggressive species and protect the surface of the metals and alloys against corrosion. Furthermore, the presence of a higher amount of Fe_3_O_4_ nanoparticles within the coating material provided a locking effect, and acted as a strong barrier at the coating–metal interface by filling the interstitial spaces and other coating artefacts (e.g., microcracks and voids), which will not allow the penetration of corrosive ions to the coating metal interface [142]. 

**Table 3:** Summary of some corrosion parameters of polymer nanocomposite coatings.

Coating	Nanomaterial	Coating Thickness (µm)	Substrate	Electrolyte	Ecorr (V vs. SCE)	Icorr (μA/cm^2^)	Corrosion Resistance	Ref.
MWCNTs-epoxy	MWCNT diameter: 2–15 nm, length: 1–10 μm, layers: 5–20	500	Steel	5% NaCl solution	No potentiodynamic test done		Charge transfer resistance after the exposure to 5% NaCl is higher for the nanocoatings than for the neat coatings for both epoxy and vinyl chloride/vinyl acetate copolymer (VYHH) resins systems.	[127]
MWCNTs-vinyl chloride/vinyl acetate copolymer		200						
Al_2_O_3_-polymer (Xylan 1810/D1864)	Al_2_O_3_ particle size 50 nm	80–100	Low carbon steel	3 wt.% NaCl solution	No values provided. Only graph		Small improvement in the corrosion resistance when 10 wt.% of Al_2_O_3_ filler were added to the polymer matrix compared to only the polymer coating, and significant improvement when compared to bare carbon steel.	[128]
No coating	GO platelet thickness: 1.3 nm, flake size: 3 µm	3.5	Carbon steel	3.5 wt.% NaCl solutions	−0.790	84.4	CS/GO-OA hydrophobic film has the lowest corrosion current and corrosion rate. Nanolayers maintained long-term anti-corrosive stability, which is correlated with hydrophobicity and permeability. Optimal filler concentration: 3 wt.% filler	[120]
CS					−0.707	18.72		
CS/GO					−0.722	15.4		
CS/GO-OA					−0.374	3.9		
GO–ZrO_2_ in EP matrix	60-nm ZrO_2_ nanoparticles and GO	65	Steel	3.5 wt.% NaCl solutions	–0.432	0.370	Well-dispersed GO–ZrO_2_ embedded in an epoxy resin (EP) matrix provided a superior barrier effect due to their two-dimensional sheet and plugging tiny pores properties Optimal filler concentration 2 wt.% of GO–ZrO_2_	[129]
SiO_2_/P(St-BA) in fluoropolymer (matrix)	10–20-nm SiO_2_ nanoparticles		Mild steel	3.5 wt.% NaCl solutions, pH 7	0.796	0.031	A 4 wt.% SiO_2_ concentration has the best corrosion resistance by increasing the barrier properties	[130]
**Conductive Polymer Nanocomposite**								
TiO_2_–polyaniline–polyvinyl butyral (PVB)	75–105-nm TiO_2_ particles	15–17	Stainless steel	3.5 wt.% NaCl solutions	No values provided. Only graph		A 100-times improvement in the corrosion resistance, especially for polyaniline prepared with 4.18 wt.% nano-TiO_2_	[136]
PVAc	5–7-nm PANI particles 16-nm ZnO particle		Stainless steel	3.5 wt.% NaCl solutions	No values provided. Only graph		After 15 days of immersion in the electrolyte, all showed a superior corrosion resistance for the hybrid coating PVAc-ZnO-Pani compared to the others	[137]
PVAc–ZnO								
PVAc–ZnO–Pani								
Graphene–polyaniline (PANI/G)	Graphene nanoflake thickness 0.569 ± 0.231	0.566 ± 0.322	Mild steel	0.1 M HCl, pH = 1	−0.532	0.572	Best corrosion resistance obtained at optimal concentration = 0.2%	[138]
CaCO_3_–polyaniline	20–56-nm CaCO_3_ nanoparticles	50	Mild steel	5 wt.% HCl solution	No potentiodynamic test done		Corrosion rate of alkyd coating is found to decrease with the increase of the polyaniline (PANI)-CaCO_3_ (PAC) nanocomposite loading in alkyd resin	[121]
				5 wt.% NaOH solution				
				5 wt.% NaCl solution	No values provided. Only graph			
no coating	_	_	Copper	5000-ppm NaCl solution	−0.331	5.2		[139]
PANI					−0.078	1.8		
PANI/G					−0.282	0.1		
**Waterborne Polymer**								
Fe_3_O_4_– epoxy acrylate (EpAc)– butylated melamine formaldehyde (BMF)	10–30-nm Fe_3_O_4_ nanoparticles	108–142	Mild steel	3.5 wt.% HCl solution	−0.694	0.215	Best corrosion resistance at 2.5 wt.% concentration of Fe_3_O_4_. Same behaviour when tested in NaCl, best resistance at 2.5 wt.% concentration	[142]
				3.5 wt.% NaOH solution	−0.222	50.8		
				Tap water	−0.512	5.343		
Fe_2_O_3_ alkyd	10–30-nm Fe_2_O_3_ nanoparticles	_	Mild steel	Salt spray	No potentiodynamic test done		A coating system with higher concentration of nano-Fe_2_O_3_ particles (0.3 wt.%) showed best corrosion resistance, UV resistance, scratch resistance, and abrasion resistance	[145]
ZnO alkyd-nano	35–40-nm ZnO nanoparticles	9–10	Mild steel	Salt spray	No potentiodynamic test done		Addition of extremely small concentration of nano-ZnO can improve the corrosion resistance, scratch resistance, and abrasion resistance of the coating	[146]

#### 2.3.2. Metallic Host Matrix Nanocomposite Coatings

Different types of nanoparticles were incorporated with nanocrystalline metal matrices coating such as silicon carbide, titanium dioxide, and alumina, to produce nanocomposite coatings [51,147,148,149,150,151,152,153,154,155]. An enhancement in corrosion resistance was reported with the addition of such nanoparticles to nickel and nickel alloy coatings. For example, in the nanocomposite coating of SiC nanoparticles with Ni, Ni–W, or Ni–Co alloys, the corrosion resistance increases as the concentration of SiC increases. This result was due to SiC nanoparticulates acting as inert physical barriers to the initiation and development of defect corrosion, as well as due to the modifying of the microstructure of the nickel layer [147,148,151,152]. Furthermore, the effect of the friction force on an SiC–Ni nanocomposite coating was examined in K_2_SO_4_ solution. Without applying any friction force, the coating maintained a stable passivation layer. However, a depassivation–repassivation process was observed on the surface of the steel at the start and at the end of applying the friction force, respectively [149].

In addition, when TiO_2_ nanoparticles were electrodeposited in composite coating with nickel over a sintered NdFeB magnet, it helped prevent the corrosive pits from growing up and accelerating the passivation process of the metal matrix as well [51]. When Al_2_O_3_ nanoparticles were incorporated into nickel coatings over steel, and tested for corrosion resistance in K_2_SO_4_ and NaCl solution, the results showed a nobler act for coated steel compared to bare steel. Al_2_O_3_ act as insulators on the composite surface, where a slightly better resistivity was found in NaCl solution [155]. Moreover, embedded Al_2_O_3_ nanoparticles in a nickel matrix refine the nickel grain and change the preferential orientation of the composite coating [154]. The type of electrodeposition coating and concentration of Al_2_O_3_ nanoparticles were found to play a key role in the corrosion behaviour study. Two types of electrodeposition techniques were used: sediment co-deposition (SCD) and adopting conventional electroplating (CEP), with different concentrations of nanoparticles. Using a CEP coating technique resulted in better corrosion resistance, which increased with the increase of Al_2_O_3_ particle concentration in the nickel matrix [154]. 

Metal nitride films are widely used as a protective layer due to their superior mechanical properties and their enhanced wear and corrosion resistances. These films are used in a binary [156] or ternary nitride [157,158,159] nanocoating system. Ternary coatings have the extra advantage of operating at high temperatures (above 700 °C), as they do not degrade into porous oxides at the film surface when used in high-temperature applications [157]. Cr–Al–N films are an example of the ternary films that are known to provide oxidation resistance up to 900 °C [158] along with high good wear resistance [159]. Adding aluminium to CrN nanocoating increases the hardness, and decreases the thermal conductivity. In addition, CrAlN films form both Al_2_O_3_ and Cr_2_O_3_ oxides layer to prevent oxygen diffusion into the bulk film at higher temperatures. However, corrosion rate values for Cr_1−*x*_Al*_x_*N coatings increased with an increase in the aluminium fraction, as the incorporation of Al in the CrN lattice increases the roughness and porosity [160]. A 0.9 ratio of Cr/Al was found to offer the best oxidation resistance when CrAlN was used to coat a 430 steel plate that was annealed with air at 800 °C [158]. Another type of binary nitride nanocoating is titanium nitride, which has been applied to gas turbine compressor blades and shown to reduce the effect of erosion [156]. The addition of Cr to this binary nanocoating improved the corrosion [161] and erosion performance, especially when it is applied in layers [162]. It was found that a CrN/AlN multilayer structure increases the corrosion performance as applying layers minimises the presence of an interconnected porosity, decreases the coating roughness, and increases the coating density [163]. Another multi-layer (nanolaminate) coating of [TiN/ZrN]_100_ deposited by a multi-arc ion plating method on the surface of Ti–6AL–4V resisted the aggressive conditions of a hot corrosion test. The mechanism of layered oxidation relieved thermal stresses and avoided the peeling that was caused by growth stress during oxidation [164]. Moreover, the further addition of silicon in CrAlSi_x_N nanocoating improved the charge transfer resistance of the surface of AISI420 stainless steel substrate when the substrate was examined with EIS testing in a 3.5 wt.% NaCl solution. According to XRD pattern and TEM images, the addition of Si retarded the columnar structure growth that is permeable to corrosive ions, and a dense coating with equiaxial grains was revealed [165]. It should be noted that Cr-based nitrides coatings are more suitable to be used at high-temperature applications than Ti-based nitrides, as the former have better strength and oxidation resistance. 

A summary for the reviewed work of this section can be found in Table 4.

##### Electroless Nickel Nanocomposite Coating

In the metal nanocomposite coating research field, nickel had received a great amount of attention due to its ability to act as the host matrix for electroless nickel plating. Electroless nickel plating is a chemical reduction process where coating is achieved by the catalytic reduction of nickel ions using a reducing agent such as sodium hypophosphite without applying electric current. Since it is a chemical reduction process, a uniform coating thickness can be obtained, along with uniform mechanical and physical properties. This was found to be an attractive and alternate method of producing a thin and uniform deposit on the substrate when compared to conventional electroplating [166,167]. The best advantage of electroless nickel coating application is to improve and create a corrosion protective layer in a highly corrosive environment [168]. 

The corrosion resistance of electroless-coated nickel phosphorous alloy is influenced by more than one factor. An increase in the corrosion resistance of the electroless nickel coating was noticed with the increase of phosphorus in the alloy. In addition, studies revealed that the incorporation of TiO_2_, Al_2_O_3_, SiC, and SiO_2_ nanoparticles into the Ni–P alloy electroless coating caused an improvement in the corrosion resistance [169,170,171,172,173,174,175]. 

The increase in corrosion resistance of the TiO_2_-Ni–P nanocoating over low carbon steel was reported to be significantly dependent on the type of the surfactant and its concentration [171]. Sodium dodecyl sulphate (SDS) as anionic surfactant and dodecyl trimethyl ammonium bromide (DTAB) as cationic surfactant were used for the deposition. A higher rate of deposition was reached with the addition of DTAB with a uniform distribution of TiO_2_. The using of DTAB at an optimum concentration to incorporate TiO_2_ in an Ni–P matrix showed the lowest corrosion rate compared with bare low carbon steel, Ni–P coating, and TiO_2_–Ni–P + SDS coating. 

In addition, heat treatment of the nanocomposite coating was found to have two opposite effects on the corrosion resistance, depending on the incorporated nanoparticles. It was reported that the nanocomposite coating of the Ni–P alloy with Al_2_O_3_ nanoparticles had an adverse effect on its corrosion resistance when heat-treated [169], while for the nanocomposite coatings of Ni–P–Zn with TiO_2_ nanoparticles and Ni–P with SiC nanoparticles, it was found to enhance corrosion resistance [172,175]. 

A summary for the reviewed work of this section can be found in Table 5.

## 3. Conclusions

Nanocoatings have significant potentials to offer superior enhancements in the corrosion performance of surfaces compared to micromaterial coatings. Nanocrystalline structures are superior over microstructures for corrosion enhancement due to the fine grain sizes, which provide better space filling and a higher integrity of the coated surface. Applying nanocoating onto the surface of the substrate makes it harder, tougher, and improves its adhesive properties. However, the coating thickness and composition should be designed so as not to decrease its protective characteristics towards corrosive and eroding influences.

Nanocoatings act through different mechanisms to provide enhanced corrosion resistance, and in some cases, they might bring up adverse effects. The fine sizes of nanocoatings form a uniform physical barrier on the surface of the material. Furthermore, nanoparticles possess improved adhesion properties due to the high density of their grain boundaries, thus increasing the corrosion resistance of the substrate. On the other hand, the higher grain boundary fraction and uneven surface generated from the agglomeration of the fine particles can foster the chance of forming anodic sites, which will make the surface more susceptible for corrosion attack. Hence, it is important to consider all of the surrounding factors related to nanocoating and substrates, in order to achieve the expected corrosion protection. 

The corrosion of metallic nanocoating has been studied with respect to the effect of several factors. It should be noted that there is no factor that affects the corrosion resistance alone in one dimension, neither one can alone contribute to the corrosion behaviour of the nanocoating. Nevertheless, they all play a role in determining the corrosion performance of the nanocoating. nanocrystalline Ni and its alloy have great potential as a promising metallic nanocoating, especially in the form of the nanocrystalline Ni–P alloy. A range of 14–17 wt.% of nickel in Zn–Ni alloys was shown to have the best corrosion resistance. The addition of phosphorus improves the corrosion behaviour in neutral and acidic media, with a composition of around 9–11 wt.% in the alloy. In regard to the grain size, there was no trend in corrosion behaviour for different sizes of the nanoparticles encountered in the nanocoating; nanocoating composition and the acidity of the media can dominate the effect of the grain size. Pulse electrodeposition was found to provide better corrosion properties than direct current, as the former technique produces a finer surface. The concentration of additives encountered in the nanocoating should be optimised for the best corrosion properties. 

The corrosion behaviour of ceramic nanocoating has been studied for different kinds of oxides, and every kind was found to have specific corrosion characteristics depending on the substrate, surroundings, and nanomaterial type and characteristics. Alumina nanocoating is shown to have enhanced corrosion characteristics when it is deposited with a plasma-enhanced ALD technique compared to thermal ALD deposition. Pre-treatment processes improves the surface properties and corrosion characteristics of the coated surface. Pre-annealing the copper substrate before coating it with Al_2_O_3_, doping TiO_2_ with nitrogen anions, or pre-etching the surface before coating it with Ta_2_O_5_ reported better corrosion resistance. Both TiO_2_ and Ta_2_O_5_ show high resistance towards corrosion in NaCl solutions. Comparing values of corrosion current densities for the studied ceramics nanocoatings in the present paper showed that the corrosion resistance of titanium oxide and tantalum oxide is higher than that for zirconia and alumina. Zirconia has the potential to replace toxic chromium in nanocoating applications, as zirconia nanocoated surfaces reveal to have fairly low corrosion current densities in various kinds of solutions. 

For nanocomposite coatings, a filler of ceramic or metallic nanoparticles is dispersed in the host matrix, which enhances the physical properties of that matrix, and enables it to be used as an effective nanocoating. Corrosion protection with nanocomposite coating is achieved by building a compact barrier and preventing charge transfer such as oxygen permeability and ion transportation. In addition, nanocomposite coating improves some of the other properties that help in enhancing the corrosion behaviour of the nanocomposite, such as: cohesive and adhesive properties, hydrophobicity, agglomeration, and dispersion and distribution properties. The corrosion of nanocomposite coating is affected by the same factors as those mentioned above. In addition to those, the corrosion behaviour of nanocomposite coatings is influenced by the nanocomposite synthesis method, type, and concentration of the filler, as well as whether the coated substrate is heat-treated or not. TiO_2_, SiC, and SiO_2_ have positive corrosion behaviour when added to the Ni–P matrix in a NaCl solution for electroless Ni coating, while for electrodeposition coating, Al_2_O_3_ showed a good corrosion resistance when blended in an Ni matrix. For polymer nanocomposite coating, conductivity has become a point of interest. 

## 4. Challenges of Corrosion Studies of the Nanocoatings

The corrosion resistance of a material defines its stability and durability, and it is important to identify it as a part of material’s performance assessment. Corrosion research provides information regarding the fundamental kinetics and mechanisms of the corrosion process. Nanocoating contains ultrafine constituents that might influence the resulting surface regarding aspects of lattice structure, grain size, porosity, intermetallic particles’ distribution, surface state, etc. These constituents have very small and dense grain boundaries that make it challenging to develop new corrosion theories for their interaction with the surface. For example, smoothening the surface increases the integrity, uniformity, and fatigue performance of the ultrathin coatings, which would decrease the possibility of pit initiation at such surfaces. At the same time, having nanoparticles covering the surface provides excessive smoothness that might weaken the adherence of the coating and cause detachments of parts of the coating. In addition, lowering the surface roughness might increase the possibility for preferential intergranular corrosion, that allows the growth of a more defective and permeable coating at the triple-junction grain boundaries. These two mechanisms allows the nanomaterials to interact with the surface in two opposite directions, and it is difficult to identify which theory is applicable. 

In addition, due to presence of such nanomaterials on the surface of the substrate, oxide formation is affected; hence, the transition mechanism of the surface state differs, and will be difficult to detect. Surface state transition from passivation to pit initiation and then to the breakdown of the film is influenced. Depending on the initial conditions of the uncoated surface and the cleanness of the final coated surface, the new state is defined, which will affect the overall surface corrosion. Due to the nonuniform distribution of the nanoparticles on the surface of the coated substrate, ions might accumulate and create weak points of higher potential that cause pit initiation. On the other hand, an accumulated coating might physically isolate the substrate surface from electrolyte ions. Predicting the transition mechanism is challenging, and it requires deeper corrosion research. 

## Figures and Tables

**Table 1:** Summary of some corrosion parameters of metallic nanocoatings.

Nanomaterial Coating	Coating Thickness	Substrate	Electrolyte	Corrosion Resistance	Tested Conditions	Ecorr (V vs. SCE)	Icorr (µA/cm^2^)	Ref.
Multi-layers of nano Cr/Cr_2_N	Individual Cr layer was 21 nm	316L Stainless steel	Artificial seawater solution	Best with the highest thickness ratio of Cr:Cr_2_N of 1.3 (lowest porosity)	Plain 316-L stainless steel	−0.59	1.23	[46]
					1.3 thickness Cr/Cr_2_N	−0.38	0.0204	
					0.18 thickness Cr/Cr_2_N	−0.51	0.0651	
NC Ni–W alloy films	30–56 µm	Mild steel	0.2 M H_2_SO_4_	Best with 100-ppm concentration of the additive	No additive	0.481	434	[47]
					50-ppm additive	−0.298	10.8	
					100-ppm additive	−0.302	7.02	
					250-ppm additive	−0.322	37.96	
NC zinc deposits (59 nm avg. grain size)	_	Without a substrate	Deaerated 0.5 N NaOH	Corrosion rate for NC zinc deposits was 60% lower than that for electrogalvanised (EG) steel samples	NC Zn	−1.47	90	[32]
					Electrogalvanised (EG) steel	−1.455	229	
NC Ni–Cu alloy (grain size 2–30 nm)	20 µm	Mild steel	Deaerated 3 wt.% NaCl Solution	Icorr values were lowest for pulse current electrodeposited Ni–Cu alloy of the 35.8 wt.% Cu and 12.7-nm avg. crystalline size	Monel-400 (67Ni-30Cu-2Fe-0.03C)	−0.314	0.807	[44]
					DC NC Ni-30.6 wt.% Cu	−0.322	0.312	
					PC NC Ni-26.0 wt.% Cu	−0.305	0.251	
					PC NC Ni-38.5 wt.% Cu	−0.294	0.113	
NC Ni–Co coating	30 µm	Carbon steel (AISI 1045)	10 w/w% NaOH solution	Addition of saccharin achieved better resistance than sodium lauryl sulphate	-	No potentiodynamic test performed. Only EIS		[65]
NC Zn–Ni alloy	_	Carbon steel	3 wt.% NaCl Solution	Best with NC Zn–Ni alloy coating of 17.62 wt.% and a 37-nm grain size.	NC Zn-12Ni alloy	−0.912	29.9	[48]
					NC Zn-17Ni alloy	−0.927	23.2	
					Microcrystalline Zn-18Ni alloy	−1.08	47.6	
NC Co and Co–P (grain sizes 67 nm and 50 nm, respectively)	15–20 µm	-	0.25-M Na_2_SO_4_ solution	Resistance order: NC Co > polycrystalline Co > NC Co–P	Nanocrystalline Co (67 grain size)	−0.574 *	0.86	[54]
					Polycrystalline Co 100 micron	−0.546 *	1.847	
NC Co–P	20 ± 2 µm	Mild steel	3.5 wt.% NaCl solution	Best with 9 wt.% P of the alloy in pulse and in direct current electrodeposition	DC-Plain Co	−0.597 *	3.3	[49]
					DC-90%Co-10%P	−0.541 *	2.0	
					DC-91%Co-9%P	−0.451 *	1.1	
					PC-91%Co-9%P	−0.476 *	0.8	
NC Co and Co-1.1 wt.% P (grain sizes 20 nm and 10 nm, respectively)	0.2 mm	Titanium	Deaerated 0.1 M H_2_SO_4_ solution	Resistance order: C Co-1.1P > microCo >~= microCo	-	No values provided. Only graph		[58]
NC Ni coating (250 nm, 54 nm, 16 nm grain size)	_	_	10 wt.% NaOH solution	Best with the lowest grain size (16 nm)	Ni 3 micon	−0.312	0.5759	[42]
					Ni 250 nm	−0.418	0.3456	
					Ni 16 nm	−0.591	0.1095	
NC Fe coating (grain size 45 nm)	8 µm	Low carbon steel	10 wt.% NaOH solution	Resistance order: NC Fe > as cast Fe > annealed Fe	NC Fe deposit	−0.35	0.289	[53]
					Annealed Fe	−0.63	5.36	
					As-cast Fe	−0.5	0.613	
NC Zn–Ni coating (grain size 28 nm with single gamma-phase)	_	Carbon Steel	3.5 wt.% NaCl solution	Best with 13 wt.% Ni content	Pure Zn	−1.039	144.2	[52]
					Zn-9.62 wt.% Ni	−0.935	52.73	
					Zn-13.31 wt.% Ni	−0.792	40.14	
					Zn-15.91 wt.% Ni	−0.826	50.99	
Ni–P (amorphous and crystalline structure)	_	Carbon Steel	3 wt.% NaCl, 1-N H_2_SO_4_, and 1-N NaOH solutions	Amorphous structure resists better than the crystalline one in neutral and acidic media, but has the same resistance in alkaline media. Higher P content had better resistance.	-	No values provided. Only graph		[57]
nano Co (67 nm grain size) and micro Co	50 µm	AISI_1045 steel	10 wt.% NaCl, 10 wt.% H_2_SO_4_, 3.5 wt.% NaCl and 0.1-M H_2_SO_4_ solutions	Icorr from highest to lowest: HCl, NaOH, NaCl, H_2_SO_4_	NC Co, 3.5 wt.% NaCl	−0.736	11.18	[43]
					NC Co, 0.1 M H_2_SO_4_	−0.343	9.837	
					NC Co, 10% NaOH	−1.022	18.91	
					NC Co, 10% HCl	−0.409	31.58	
NC Cu-70Zr (10–20 nm grain size)	20 µm	_	Deaerated 0.1-M and 0.5-M HCl solutions	Grain refinement has a stabilisation effect on the corrosion process	-	No values provided. Only graph		[45]

NC: nanocrystalline. Voltage measured vs. Ag/AgCl. EIS: electron impedance spectroscopy.

**Table 4:** Summary of some corrosion parameters for metallic host matrix nanocomposite coatings.

Coating	Nanomaterial (Particle Size in nm)	Coating Thickness (µm)	Substrate	Electrolyte	Ecorr (V vs. SCE)	Icorr (μA/cm^2^)	Corrosion Resistance	Ref.
Al_2_O_3_–Ni	Al_2_O_3_ (13)	50	Steel	0.5 M potassium and sulphate solution	−0.1588	0.5		[155]
				0.5 M NaCl solution	−0.3592	0.43		
Al_2_O_3_–Ni	Al_2_O_3_ (100)	25	Mild steel	3.5 wt.% NaCl solutions	−0.253	0.011	Highest value with sediment co-deposition technique (SCD) at 7.58 wt.% Al_2_O_3_	[154]
Al_2_O_3_–Ni	Al_2_O_3_ (40)	_	Steel	0.5 M Na_2_SO_4_ solution	−0.150	1.42		[147]
SiC–Ni	SiC (45)				−0.170	2.81		
Al_2_O_3_ + SiC–Ni	Al_2_O_3_ + SiC (40–45)				−0.130	1.02		
SiC–Ni	SiC (50)	_	Copper	3.5 wt.% NaCl solution	No values provided. Only graph			[148]
SiC–Ni	SiC (20)	50		0.5 M K_2_SO_4_ solution	No values provided. Only graph			[149]
SiC–Ni	SiC (20)	200	Carbon-steel	0.5 M Na_2_SO_4_	−0.2605	1.9		[150]
SiC–Ni	SiC (40)		Copper	3.5 wt.% NaCl solution	−0.248	0.6645		[151]
SiC–Ni–W	SiC (80)		Copper	3.5 wt.% NaCl solution	No values provided. Only graph		-	[152]
SiC–Ni–Co	SiC (50)	20	Copper	3.5 wt.% NaCl solution	-	7900	Highest at 3.2 wt.% of SiC in Ni-Co matrix	[153]
TiO_2_–Ni	TiO_2_ (10)	_	Sintered NdFeB magnet	3.5 wt.% NaCl solution	-	0.214	-	[51]

**Table 5:** Summary for some corrosion parameters of electroless nickel alloy nanocomposite coatings.

Coating	Nanomaterial (Particle Size in nm)	Coating Thickness (µm)	Substrate	Electrolyte	Ecorr (V vs. SCE)	Icorr (μA/cm^2^)	Corrosion Resistance	Ref.
Al_2_O_3_–Ni–P	Al_2_O_3_ (50)	8–12	Low carbon steel	3.5 wt.% NaCl solution	No values provided. Only graph		The highest surface resistance was with the 75 g/l alumina (Al3) coatings (100 times higher than the as-polished samples). The surface resistance decreased sharply after heat treatment.	[169]
TiO_2_–Ni–P	TiO_2_ (30–60)	0.038	Copper	3.5 wt.% NaCl solution	−0.26	0.34	Optimum conditions: concentration of nickel source solution: 50 g/L, concentration of reducing agent: 10 g/L, concentration of TiO_2_ powder: 10 g/L, and bath temperature: 85 °C	[170]
TiO_2_–Ni–P	TiO_2_ (25)	_	Low carbon steel	3.5 wt.% NaCl solution	−0.318	5.38	The corrosion resistance was the highest with 4.347 g/l concentrations of dodecyl trimethyl ammonium bromide (DTAB) surfactant, with 86.13 wt.% Ni, 6.92 wt.% P, and 6.95 wt.% TiO_2_	[171]
TiO_2_–Ni–Zn–P	TiO_2_ (100–200)	_	Low carbon steel	3.5 wt.% NaCl solution	−0.404	0.364	Highest corrosion resistance for heat-treated coating at 6.18 wt.% Zn, 10.56 wt.% P, and 2.30 wt.% TiO_2_	[172]
SiC–Ni–P	SiC (40)	_	X70 steel	CO_2_ containing media in the presence of acetic acid	−0.440	1.1	Optimum concentration is 2.45 wt.% of SiC in the coating	[173]
SiO_2_–Ni–P	SiO_2_ (20)	29	API-5L X65 steel substrates	3.5 wt.% NaCl solution	−0.336	0.308	Optimum concentration at 2 wt.% of SiC	[174]
SiC–Ni–P	SiC (40)	20 ± 1	St37 tool steel substrate	3 wt.% NaCl solution	−0.255	1.58	Highest corrosion resistance for heat-treated nanocomposite at 4 wt.% of SiC in Ni–P	[175]
